# Epithelial p38α Controls Immune Cell Recruitment in the Colonic Mucosa

**DOI:** 10.1371/journal.ppat.1000934

**Published:** 2010-06-03

**Authors:** Young Jun Kang, Motoyuki Otsuka, Arjen van den Berg, Lixin Hong, Zhe Huang, Xiurong Wu, Duan-Wu Zhang, Bruce A. Vallance, Peter S. Tobias, Jiahuai Han

**Affiliations:** 1 Department of Immunology and Microbial Science, The Scripps Research Institute, La Jolla, California, United States of America; 2 The Key Laboratory of the Ministry of Education for Cell Biology and Tumor Cell Engineering, School of Life Sciences, Xiamen University, Xiamen, Fujian, China; 3 Division of Gastroenterology, BC Children's Hospital, Vancouver, British Columbia, Canada; University of Toronto, Canada

## Abstract

Intestinal epithelial cells (IECs) compose the first barrier against microorganisms in the gastrointestinal tract. Although the NF-κB pathway in IECs was recently shown to be essential for epithelial integrity and intestinal immune homeostasis, the roles of other inflammatory signaling pathways in immune responses in IECs are still largely unknown. Here we show that p38α in IECs is critical for chemokine expression, subsequent immune cell recruitment into the intestinal mucosa, and clearance of the infected pathogen. Mice with p38α deletion in IECs suffer from a sustained bacterial burden after inoculation with *Citrobacter rodentium*. These animals are normal in epithelial integrity and immune cell function, but fail to recruit CD4^+^ T cells into colonic mucosal lesions. The expression of chemokines in IECs is impaired, which appears to be responsible for the impaired T cell recruitment. Thus, p38α in IECs contributes to the host immune responses against enteric bacteria by the recruitment of immune cells.

## Author Summary

The cellular responses of intestinal epithelial cells (IECs) to microorganisms in the gastrointestinal tract are mediated by activation of a number of intracellular signaling pathways. It was shown that the NF-κB pathway in IECs is essential for epithelial integrity and intestinal immune homeostasis, and here we show that p38α-mediated signaling in IECs is not important for epithelial integrity and immune cell function, but is critical for the clearance of the infected pathogen. p38α in IECs is essential for pathogen-induced chemokine expression in IECs and for subsequent immune cell recruitment into the intestinal mucosa, which leads to the clearance of the infectious pathogen. Our results indicate that different intracellular signaling pathways in IECs mediate distinct cellular responses to microorganisms in the gastrointestinal tract, and this information should be taken into consideration in the development of pathway-targeted therapeutic interventions for gastrointestinal infection.

## Introduction

Attaching and effacing (A/E) bacterial pathogens, such as the enteropathogenic *Escherichia coli* (EPEC) and enterohemorrhagic *E. coli* (EHEC), cause debilitating disease, especially among infants and children, and are a threat to global health [1], [2]. *Citrobacter rodentium* (*C. rodentium*) is an A/E pathogen, which occurs naturally in mice, and serves as an excellent animal model for these mucosal infections [3], [4]. *C. rodentium* has a remarkable ability to colonize the murine colon and cecum, but is typically subclinical and self-limiting, and is eventually cleared from the gastrointestinal tracts in immunocompetent mice [3]. Studies of *C. rodentium* infection in immunodeficient mice have established that CD4^+^ T cells and *C. rodentium*-specific antibody responses are essential components of adaptive immunity for eradicating the infection [5], [6], and recent studies have revealed that T_H_1 and T_H_17 immune responses have important host defense functions during *C. rodentium* infection [7]–[9]. However, the molecular mechanism by which these immune responses are regulated after the mucosal surface of the intestinal tract is stimulated by pathogens is still largely unknown.

The role of the NF-κB pathway in intestinal epithelial cells was reported recently using IKK subunit knockout mice [10], [11]. The NF-κB pathway in intestinal epithelial cells is essential for intestinal immune homeostasis, although the mechanisms are not exactly the same, as one study reported dysregulated epithelial cell integrity while another reported dysregulated immune cell function after different pathogen infections [10], [11]. These results tempted us to explore the role of p38α, another major inflammatory pathway, in intestinal epithelial cells and its role in immunity to enteric pathogens.

p38α is the prototypic member of the p38 group of mitogen-activated protein kinases (MAPKs) [12], and its activation has a pivotal role in linking inflammatory stimuli to cellular responses [13]–[15]. Previous studies using a human colon epithelial cell line (Caco-2) have shown a role for p38α in enteric pathogen-induced IL-8 production [16], but the role of p38α in intestinal epithelial cells *in vivo* is not known. The embryonic lethality of p38α-null mice and the limited target specificity of p38 inhibitors on p38α are limiting factors for understanding the role of p38α *in vivo*. Here we used *C. rodentium* infection and mice lacking p38α in intestinal epithelial cells to study the role of p38α in host responses to mucosal infection. We found that unlike the NF-κB pathway, which controls intestinal immune homeostasis, intestinal epithelial p38α is crucial for immune cell recruitment in the colonic mucosa. The different inflammatory signaling pathways appear to differentially affect immune responses in intestinal epithelial cells.

## Results

### p38α in intestinal epithelial cells is involved in immunity to *C. rodentium*

**Figure 1:**
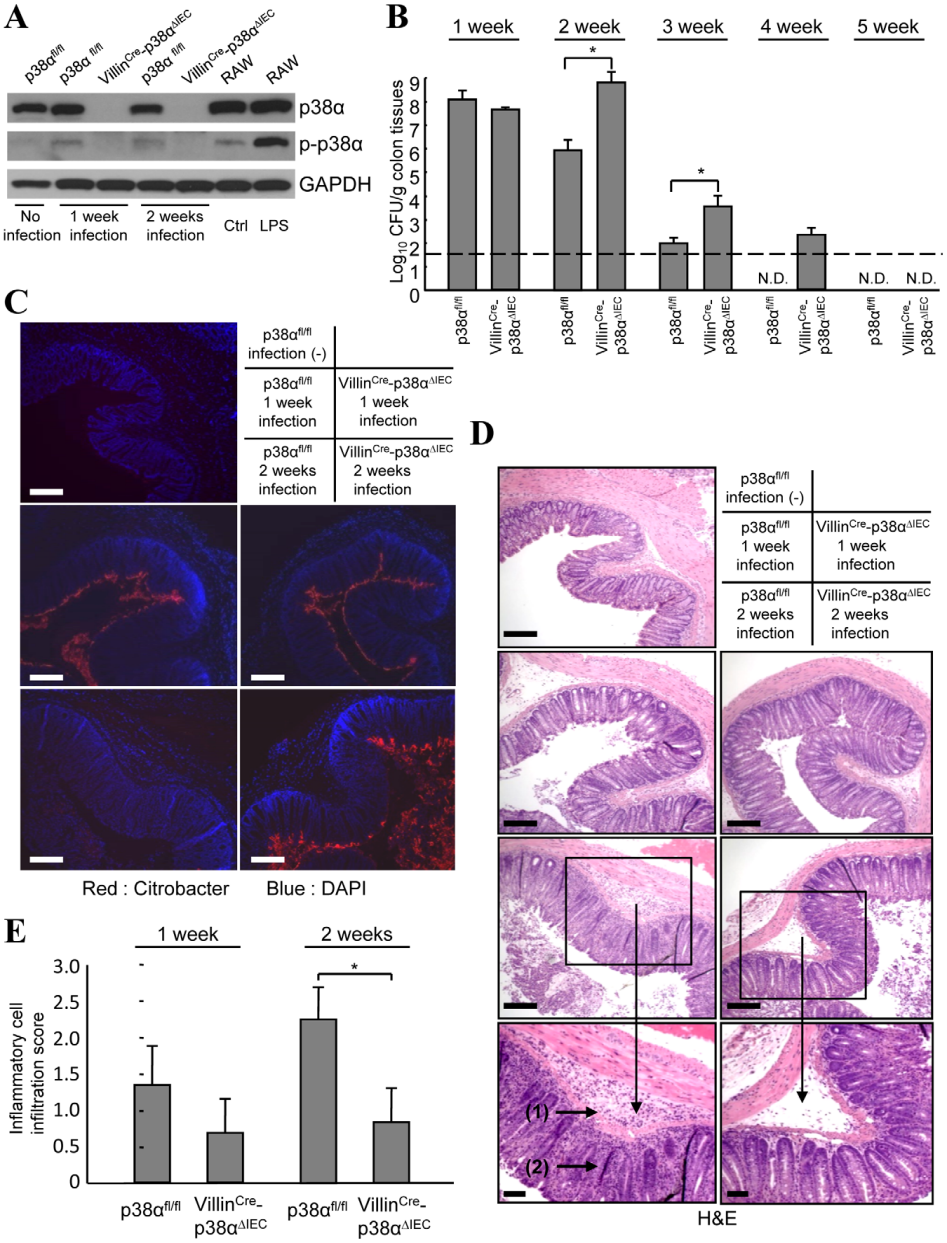
p38α in intestinal epithelial cells is required for the immunity to *C. rodentium*. **A**, The level of p38α and phosphorylated-p38α (p-p38α) in isolated IECs from non-infected mice (lane 1), or p38α^fl/fl^ and Villin^Cre^-p38α^ΔIEC^ mice infected with *C. rodentium* for 1 or 2 weeks (lane 2, 4 and 3, 5). RAW264.7 cells, a mouse macrophage cell line, treated with or without LPS for 1 hour were used as controls for p38α phosphorylation (lane 6, 7). The data is representative from two independent experiment sets. **B**, *C. rodentium* CFU recovered from colon tissues of individual p38α^fl/fl^ and Villin^Cre^-p38α^ΔIEC^ mice at 1, 2, 3, 4 and 5 weeks after inoculation. The data shown are from one experiment, representative of five. The transverse bar is the detection limit. Asterisk, p<0.05. Error bars indicate s.d. (*n* = 6). N.D. not detected. **C**, Immunofluorescence staining of *C. rodentium* in the colon segments by anti-*C. rodentium* antibodies (red). Nuclei were counterstained with DAPI (blue). Colon segments isolated from a non-infected p38α^fl/fl^ control mouse, and from p38α^fl/fl^ or Villin^Cre^-p38α^ΔIEC^ mice at 1 and 2 weeks after infection were used. Scale bar, 100 µm. **D**, Inflammation detected by hematoxylin/eosin staining of colon segments. The sections are adjacent to those in C. Scale bar, 100 µm. **E**, The inflammatory cell infiltration into the submucosa in 1 or 2 weeks infected p38α^fl/fl^ or Villin^Cre^-p38α^ΔIEC^ mice was evaluated by histological score.

**Figure 2:**
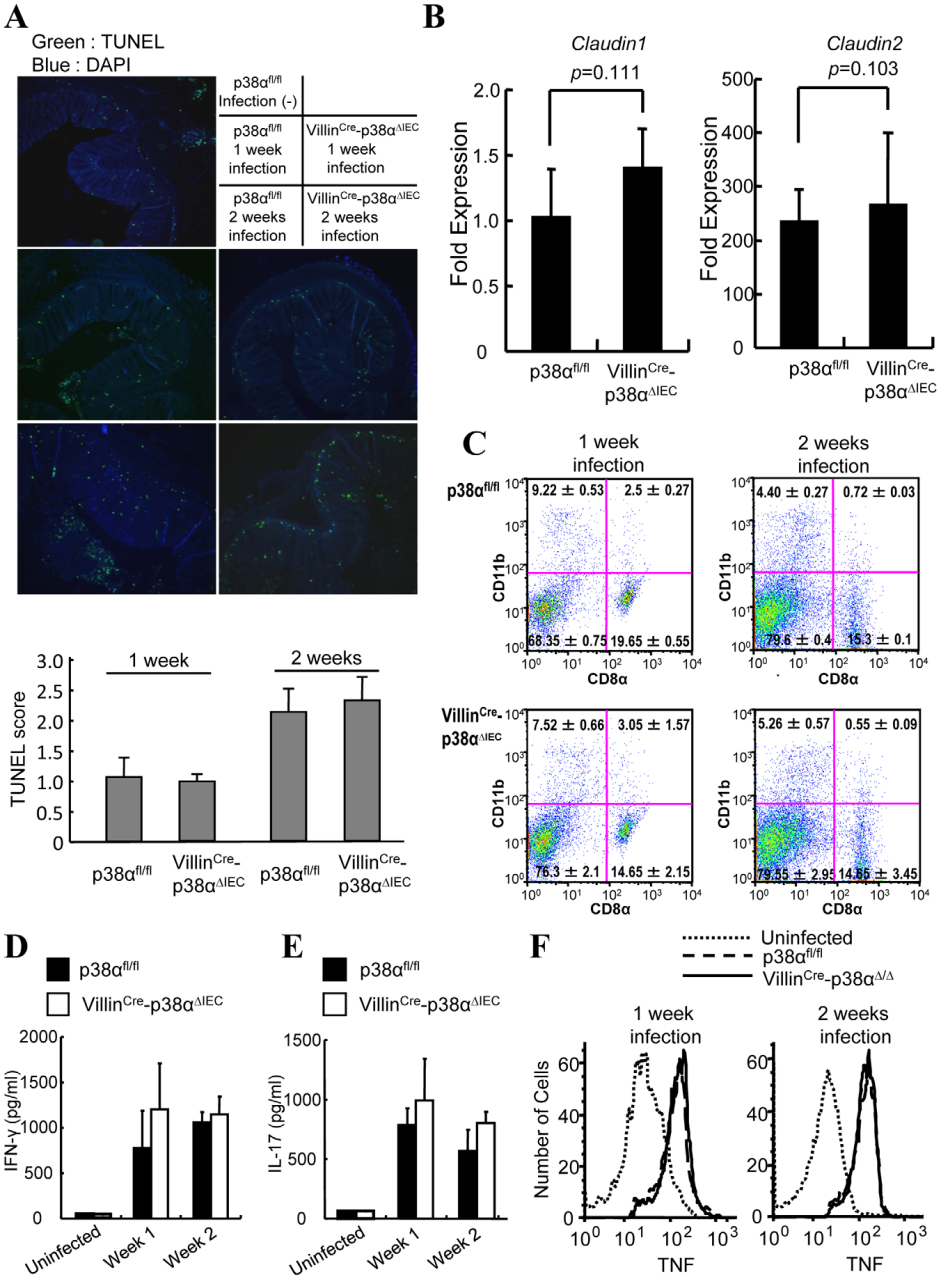
Epithelial integrity and immune cells are normal in Villin^Cre^-p38α^ΔIEC^ mice after *C. rodentium* infection. **A**, TUNEL staining (green) on colon cross sections from non-infected p38α^fl/fl^ control and p38α^fl/fl^ or Villin^Cre^-p38α^ΔIEC^ mice at 1 and 2 weeks after infection. Scale bar, 100 µm. The sections were adjacent to those in Fig. 1C. **B**, Expression of tight junction proteins in the intestinal epithelial cells of *C. rodentium*-infected mice. Expression of Claudin-1 and -2 was measured by qPCR method. Fold expression of proteins was calculated over the expression of proteins of uninfected IECs. GADPH levels was measured as an internal control. **C**, Composition of the dendritic cell compartment in the mesenteric lymph node cells of p38α^fl/fl^ or Villin^Cre^-p38α^ΔIEC^ mice at 1 and 2 weeks after infection. **D & E**, *C. rodentium*-specific IFN-γ (**D**) and IL-17 (**E**) responses after restimulation of mesenteric lymph node cells from p38α^fl/fl^ or Villin^Cre^-p38α^ΔIEC^ mice at 1 and 2 weeks after infection. Data are representative of three independent experiments (*n* = 3). Cells from uninfected mice were used as a control. **F**, Expression of TNF-α by the macrophages (F4/80 positive cells) of the draining mesenteric lymph nodes of p38α^fl/fl^ or Villin^Cre^-p38α^ΔIEC^ mice at 1 and 2 weeks after infection. Data are representative of two independent experiments (*n* = 3).

*C. rodentium* is a popular surrogate mouse model for the study of attaching and effacing bacterial pathogens. Their attachment to mouse colonic epithelial cells results in effacement of the brush border, termed an A/E lesion, and colonic mucosal hyperplasia [17]. To investigate the function of p38α in the intestinal epithelium, we generated mice lacking p38α in intestinal epithelial cells (Villin^Cre^-p38^ΔIEC^) by crossing *loxP*-flanked (p38α^fl/fl^) mice with villin-Cre (Villin^Cre^)-expressing mice. These mice appear healthy and have no remarkable histological abnormalities in the intestine, currently studied up to seven months after birth. Lack of p38α protein in the intestinal epithelial cells from Villin^Cre^-p38^ΔIEC^ mice was confirmed by immunoblotting (Fig. 1A). *C. rodentium* infection induced p38α phosphorylation in the intestinal epithelial cells of p38α^fl/fl^ mice (Fig. 1A), indicating an involvement of p38α in the *C. rodentium*-induced host response. *C. rodentium* inoculation induced rapid and transient body weight loss in both p38α^fl/fl^ and Villin^Cre^-p38^ΔIEC^ mice; however, Villin^Cre^-p38^ΔIEC^ showed impaired body weight recovery after 7 days of infection (Supplementary Fig. S1). The difference between wildtype and Villin^Cre^-p38^ΔIEC^ mice was moderate but statistically significant (Supplementary Fig. S1). We further analyzed bacterial burden in the colon tissues of p38α^fl/fl^ and Villin^Cre^-p38^ΔIEC^ mice and found it to be comparable at the early times of infection, but much worse in Villin^Cre^-p38^ΔIEC^ mice after two weeks of infection (Fig. 1B and Supplementary Fig. S2). Moreover, the eventual clearance of the bacteria occurred later in Villin^Cre^-p38^ΔIEC^ mice (Fig. 2B), indicating that Villin^Cre^-p38^ΔIEC^ mice exhibit a significant defect in clearing bacteria from the colon tissues. Immunohistological studies showed that at 1 week after infection, *C. rodentium* localized close to the surface of the colon epithelial cells similarly in p38α^fl/fl^ and Villin^Cre^-p38^ΔIEC^ mice (Fig. 1C). However, at two weeks after infection, p38α^fl/fl^ mice showed only a slight bacterial staining on the colon surfaces, whereas numerous *C. rodentium* still remained in Villin^Cre^-p38^ΔIEC^ mice (Fig. 1C). The greater bacterial burden recovered from the colons of Villin^Cre^-p38^ΔIEC^ mice two-weeks after infection was confirmed by qPCR to quantify bacterial 16s rDNA (Supplementary Table S1). H&E staining using adjacent sections showed inflammatory cell invasion into the colonic mucosa at two weeks after infection (Fig. 1D). However, the degree of inflammatory cell infiltration was more severe in p38α^fl/fl^ mice two weeks after infection (Fig. 1D and 1E), but the bacterial burden was less in those mice compared with the Villin^Cre^-p38^ΔIEC^ mice (Fig. 1B and 1C). These results indicate that p38α in intestinal epithelial cells is involved in the clearance of infected *C. rodentium*, and the role of epithelial p38α in inflammatory cell invasion is at least part of the underlying mechanism.

### Epithelial integrity and functions of mesenteric lymph node immune cells are normal in Villin^Cre^-p38^ΔIEC^ mice after *C. rodentium* infection

NF-κB signaling, a well-known major inflammatory pathway, has been explored in the gut recently [10], [11]. Deletion of IκB kinase-β (IKKβ) or IKKγ in intestinal epithelial cells causes abnormal epithelial integrity and subsequent abnormal spontaneous inflammation [11] or impaired conditioning of dendritic cells and subsequent impaired T cell polarization after parasite inoculation [10]. Here we examined the epithelial integrity and immune cell functions in our Villin^Cre^-p38^ΔIEC^ mice. TdT-mediated dUTP nick end labeling (TUNEL) staining of colon tissues before and after *C. rodentium* infection revealed no significant differences in epithelial cell viability between p38α^fl/fl^ and Villin^Cre^-p38^ΔIEC^ mice (Fig. 2A and data not shown), although more intestinal epithelial cells, especially located at the bottom of the mucosa, showed evidence of apoptosis after *C. rodentium* infection in both animals (Fig. 2A). Expression of claudin-1 and claudin-2, members of the tight junction protein family that regulate epithelial permeability, were analyzed by qRT-PCR using colon tissues from *C. rodentium*-infected and uninfected mice. As reported [18], [19], claudin-2 was strongly induced while claudin-1 expression was not affected by *C. rodentium*-infection (Fig. 2B). Deletion of p38α in intestinal epithelial cells did not affect claudin-1 and claudin-2 expression as compared to infected and uninfected p38α^fl/fl^ mice (Fig. 2B). This data supports the conclusion that intestinal epithelial integrity is not affected by p38α deletion. Consistently, we did not detect bacterial translocation into the colon mucosa in either p38α^fl/fl^ or Villin^Cre^-p38^ΔIEC^ mice by IHC (Figure 1C and Supplementary Fig. S3, the adjacent sections were used in Figure 1C, S3, 2A, and 1D) and by qRT-PCR to quantify bacterial 16s rDNA (data not shown).

We then analyzed immune cells in the mesenteric lymph nodes, as these nodes drain the murine large bowel, potentially in concert with the caudal lymph nodes. The size of mesenteric lymph nodes was similar in p38α^fl/fl^ and Villin^Cre^-p38^ΔIEC^ mice. To examine dendritic cells (DC), the composition and function of DC subsets in the intestine-associated lymphoid tissue of p38α^fl/fl^ and Villin^Cre^-p38^ΔIEC^ mice was examined after *C. rodentium* infection. Similar frequencies of CD11c^+^CD11b^−^CD8α^−^ (double-negative, DN), CD11c^+^CD11b^−^CD8α+ (CD8α^+^), and CD11c^+^CD11b^+^CD8α^−^ (CD11b^+^) DC subsets were observed in mesenteric lymph node cells of p38α^fl/fl^ and Villin^Cre^-p38^ΔIEC^ mice at one and two weeks after *C. rodentium* infection (Fig. 2C). No significant difference in tumor necrosis factor (TNF)- α production by the CD11c^+^CD11b^+^CD8α^−^ (CD11b^+^), CD11c^+^CD11b^−^CD8α^+^ (CD8α^+^), or CD11c^+^CD11b^−^CD8α^−^ (double negative) DC subset population of the draining mesenteric lymph nodes was observed (Supplementary Fig. S4A, S4B, S4C). In addition, no T cell functional polarization shift was observed, as similar amounts of IFN-γ (T_H_1 response) and IL-17 (T_H_17 response) were produced after bacterial antigen stimulation of draining mesenteric lymph node cells of p38α^fl/fl^ and Villin^Cre^-p38^ΔIEC^ mice at 1 and 2 weeks after *C. rodentium* infection (Fig. 2D, 2E). In supporting this notion, the CD4^+^T cells from mesenteric lymph node or lamina propria of *C. rodentium*-infected p38α^fl/fl^ and Villin^Cre^-p38^ΔIEC^ mice showed similar expression of IFN-γ and IL-17 (Supplementary Fig. S5 and S6). In addition, the production of TNF by macrophages in draining mesenteric lymph nodes was also comparable in the p38α^fl/fl^ and Villin^Cre^-p38^ΔIEC^ mice (Fig. 2F). These results indicate that, unlike the ablation of the NF-κB pathway in intestinal epithelial cells, epithelial integrity and immune cell in the intestine-associated lymphoid tissue are normal in Villin^Cre^-p38^ΔIEC^ mice.

### p38α in intestinal epithelial cells is required for T cell recruitment into the colon mucosa after *C. rodentium* infection

**Figure 3:**
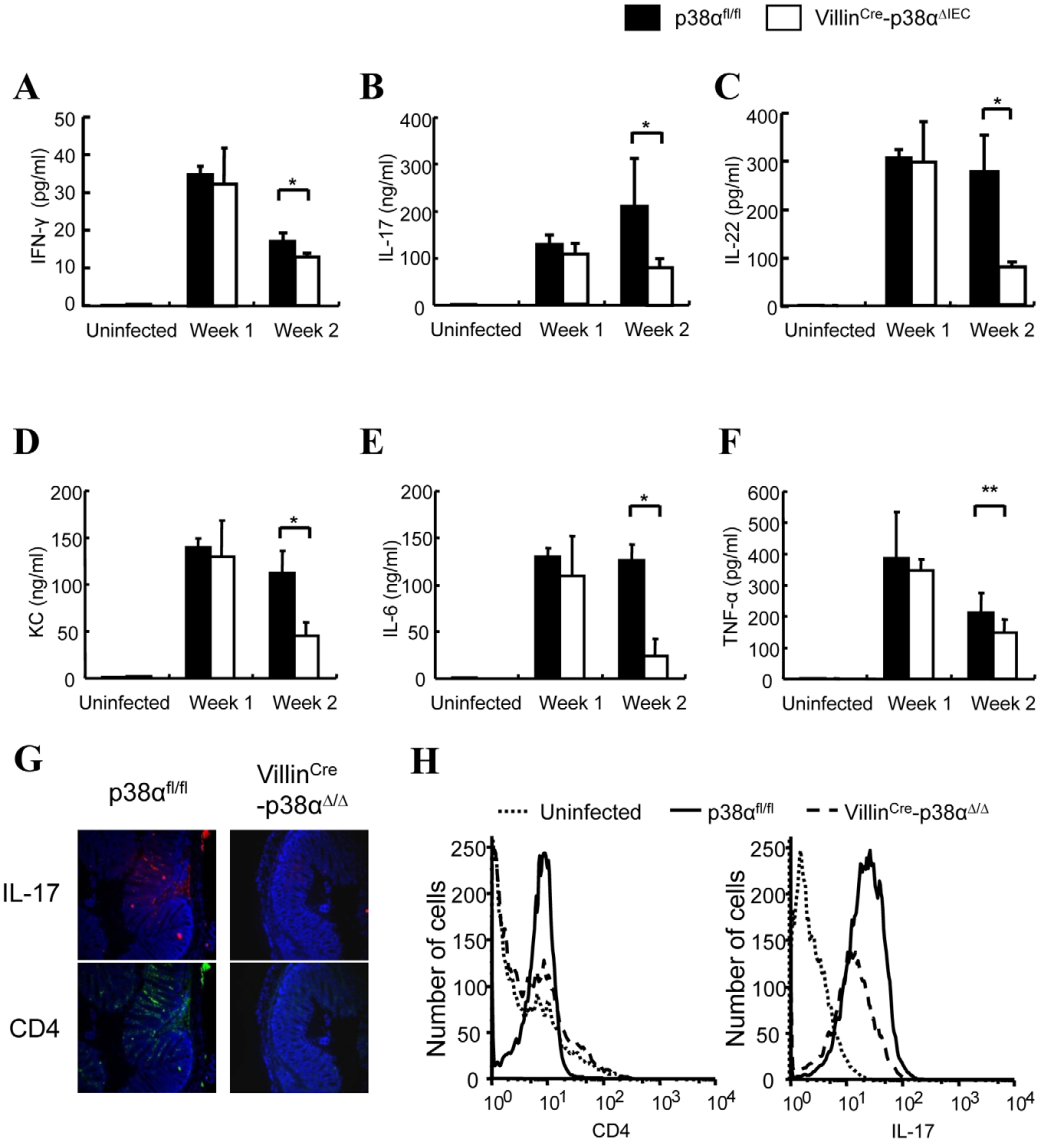
Cytokine expressions in the colon of VillinCre-p38α^ΔIEC^ mice are impaired after *C. rodentium* infection. **A–F**, *ex vivo* colon culture ELISA of IFN-γ, IL-17, IL-22, KC, IL-6, and TNF expression in p38α^fl/fl^ or Villin^Cre^-p38α^ΔIEC^ mice colons at 1 and 2 weeks after *C. rodentium* infection. Asterisk, p<0.05; Double asterisk, p>0.05. Error bars indicate s.d. Data are representative of two independent experiments (*n* = 3). **G & H**, Expression of IL-17 in the colon mucosa of p38α^fl/fl^ or Villin^Cre^-p38α^ΔIEC^ mice at 2 weeks after *C. rodentium* infection, determined by immunofluorescent staining for CD4 (green) and IL-17 (red), and nuclei were counterstained with DAPI (blue) (**G**), and by FACS analysis of whole colon cells using anti-CD4-PE and IL-17-APC antibodies (**H**). The basal levels of CD4 and IL-17 of uninfected p38α^fl/fl^ colon cells are shown in the FACS analysis.

Although the functions of immune cells isolated from mesenteric lymph nodes and lamina propria of Villin^Cre^-p38^ΔIEC^ mice appeared to be normal when compared with that of p38α^fl/fl^ mice, the expression of various T_H_1 and T_H_17 related cytokines in the colon, including IFN-γ, IL-17, and IL-22, which are critical factors in the host defense against A/E bacterial pathogens [9], [20], was significantly less in Villin^Cre^-p38^ΔIEC^ mice two weeks after *C. rodentium* infection (Fig. 3A, B, C). Similar expression patterns of KC and IL-6, but not TNF, were also observed (Fig. 3D, E, F). Immune cells in colon include resident cells in lamina propria and infiltrated cells in the colonic mucosa. Because there is no functional difference between the immune cells isolated from lamina propria of Villin^Cre^-p38^ΔIEC^ and p38α^fl/fl^ mice (Supplementary Fig. S6), the differences shown in Fig. 3E are likely caused by infiltrated immune cells. Therefore, we determined whether deletion of p38α in IEC affects the number of infiltrated T_H_17 cells in colon. Immunostaining showed that there were more infiltrated T_H_17 cells in the colons of p38α^fl/fl^ mice in comparison with that of Villin^Cre^-p38^ΔIEC^ mice, and the majority of T_H_17 cells were CD4^+^ cells (Fig. 3G). Flow cytometry analysis of cells isolated from colon tissues confirmed this result (Fig. 3H).

**Figure 4:**
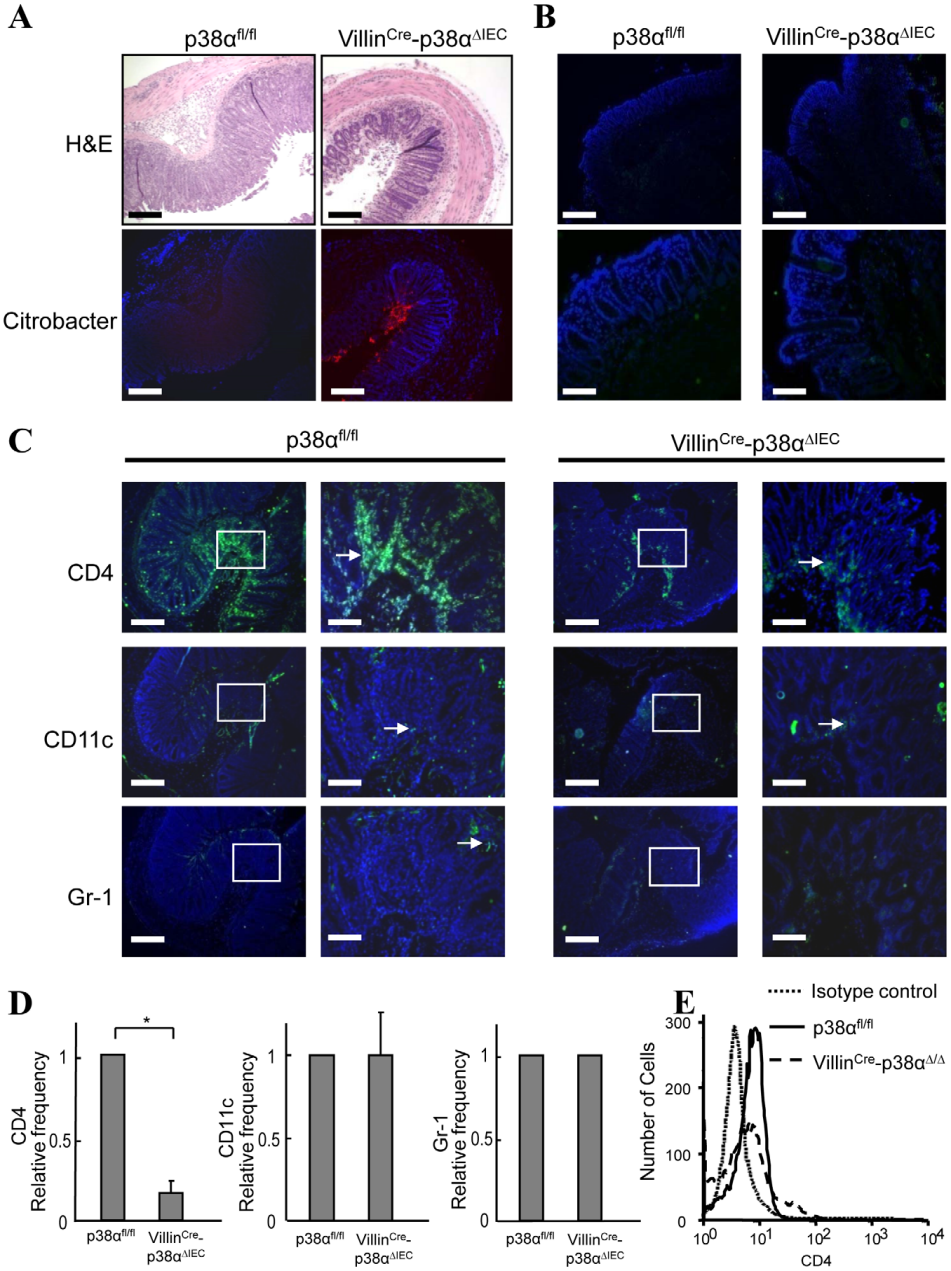
p38α in intestinal epithelial cells is required for chemokine expression and to recruit immune cells into the colon mucosa after *C. rodentium* infection. **A,** Histological changes with inflammation (upper) by hematoxylin/eosin staining and the immunofluorescence staining of *C. rodentium* (lower; red) in the adjacent distal colon cross sections from p38α^fl/fl^ or Villin^Cre^-p38α^ΔIEC^ mice at 2 weeks after infection. Scale bar, 100 µm. **B,** CD4^+^ T cell infiltration in the distal colon mucosa of non-infected p38α^fl/fl^ or Villin^Cre^-p38α^ΔIEC^ mice, determined by immunofluorescent staining (green). Scale bar, 100 µm (low magnification; upper panels), 30 µm (high magnification; lower panels). Nuclei were counterstained with DAPI (blue). **C,** Cell infiltration in the distal colon mucosa of p38α^fl/fl^ or Villin^Cre^-p38α^ΔIEC^ mice at 2 weeks after infection, determined by immunofluorescent staining for CD4, CD11c, and Gr-1 (green). Right panels are enlarged images of the parts denoted in the boxes in the left panels in each group. Scale bar, 100 µm (low magnification), 30 µm (high magnification). **D,** Relative frequencies of CD4+, CD11c+, and Gr-1+ cells in the distal colon mucosa from infected Villin^Cre^-p38α^ΔIEC^ mice in comparison with that from infected p38α^fl/fl^ mice. Immunostained cells were counted in total ten fields of each mouse. The relative frequencies were calculated after adjusting the number from infected p38α^fl/fl^ mice as 1. The results show mean ± s.d. from four animal pairs. Data are representative of 2–4 independent experiments. Asterisk, p<0.05. **E,** Infiltration of CD4^+^ T cells to the lamina propria. Lamina propria lymphocytes were obtained from p38α^fl/fl^ or Villin^Cre^-p38α^ΔIEC^ mice at 2 weeks after infection, and stained with anti-CD3-FITC and anti-CD4-PE antibodies for FACS analysis. The number of CD4^+^ cells is shown in the CD3^+^ T cell population. 5000 cells were analyzed per sample.

Since the degree of inflammatory cell infiltration was less severe in Villin^Cre^-p38^ΔIEC^ mice in comparison with p38α^fl/fl^ mice (Fig. 1D, 1E and 4A), the infiltration of immune cells into the colonic mucosa was examined in more detail. Various frozen sections of colon were stained with antibodies against CD4, CD11c (as a DC marker), Gr-1 (as a neutrophil marker), and F4/80 (as a macrophage marker, data not shown). No staining of any marker used here was detected in the colon tissues of either p38α^fl/fl^ and Villin^Cre^-p38^ΔIEC^ mice before *C. rodentium* infection (Fig. 4B and data not shown), and a few scattered immune cells that had infiltrated into the mucosa were detected at 1 week after infection (Supplementary Fig. S7). Infiltration of immune cells, especially CD4^+^ T cells, was dramatically increased in p38α^fl/fl^ mice two weeks after infection (Fig. 4C), consistent with previous reports that CD4^+^ T cells infiltrate into the colonic mucosa and play a central role in the clearance of this bacterium [5]. In contrast, the infiltration of CD4^+^ T cells into the colonic mucosa of Villin^Cre^-p38^ΔIEC^ mice two weeks after *C. rodentium* infection was much less than that in p38α^fl/fl^ mice (Fig. 4C, 4D and Supplementary Fig. S8). FACS analysis of CD4^+^T cells in isolated lamina propria cells confirmed the decreased CD4^+^T cell infiltration into the colonic mucosa in Villin^Cre^-p38^ΔIEC^ mice (Fig. 4E). These results suggest that p38α in intestinal epithelial cells is required for the CD4^+^ T cell recruitment into the colonic mucosa.

### p38α is required for chemokine expression in intestinal epithelial cells, which is essential for the recruitment of immune cells into the colonic mucosa after *C. rodentium* infection

**Figure 5:**
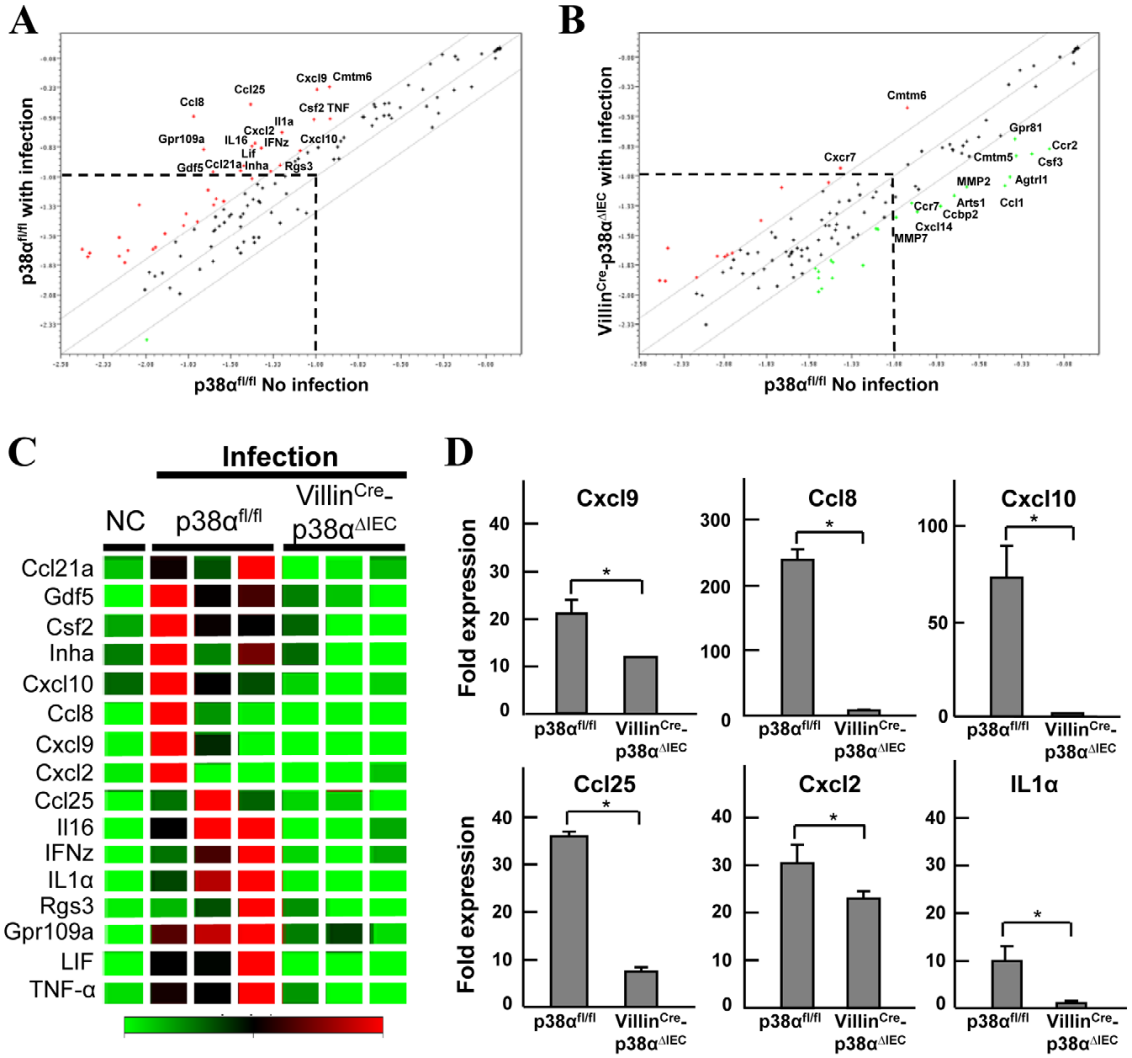
p38α in intestinal epithelial cells is required for chemokine expression to recruit immune cells into the colon mucosa after *C. rodentium* infection. **A, B**, Linear scatter plot of gene expression. Each gene in the microarray is represented by a point with coordinates consisting of average gene expression in log scale (*n* = 3) from isolated IECs of p38α^fl/fl^ (**A**) or Villin^Cre^-p38α^ΔIEC^ (**B**) mice at 1 week after infection, in comparison with those of a non-infected p38α^fl/fl^ mouse. The genes with more than a 2 fold increase or less than a 0.5 fold decrease are represented as red or green, respectively. Gene names are indicated only if those expression levels are higher than a threshold and significantly changed. **C**, The genes determined as induced more than 2 fold in the IECs of infected p38α^fl/fl^ mice in (a) are selectively shown in the clustering image. **D**, Quantitative RT-PCR of gene expression in isolated IECs of p38α^fl/fl^ or Villin^Cre^-p38α^ΔIEC^ mice at 1 week after infection. Values represent amounts relative to that of uninfected p38α^fl/fl^ mouse samples. Asterisk, p<0.05. Error bars indicate s.d. Data is representative of three experiments.

Because p38α is important for cytokine expression [13]–[15], the expression of chemokines in the colonic epithelial cells of p38α^fl/fl^ and Villin^Cre^-p38^ΔIEC^ mice one week after *C. rodentium* infection was determined by microarray analysis (Fig. 5A, B, and C). The time point was chosen as the time when the bacterial burden is still similar between p38α^fl/fl^ and Villin^Cre^-p38^ΔIEC^ mice and when immune cells start to infiltrate into the colonic mucosa as described above. Although Cmtm6 was upregulated similarly in both p38α^fl/fl^ and in Villin^Cre^-p38^ΔIEC^ mice after infection, the expression of most genes in p38α^fl/fl^ mice colon epithelial cells that were upregulated by infection did not change in Villin^Cre^-p38^ΔIEC^ mice colon epithelial cells (Fig. 5A, B and C). Rather, the expression of some genes was downregulated, even after infection, compared with those in uninfected p38α^fl/fl^ control mice colon epithelial cells (Fig. 5B). We confirmed the differential expression of the selected genes by qRT-PCR (Fig. 5D), finding that p38α deletion indeed reduced the expression of a number of *C. rodentium* infection-induced chemokines in colon epithelial cells.

**Figure 6:**
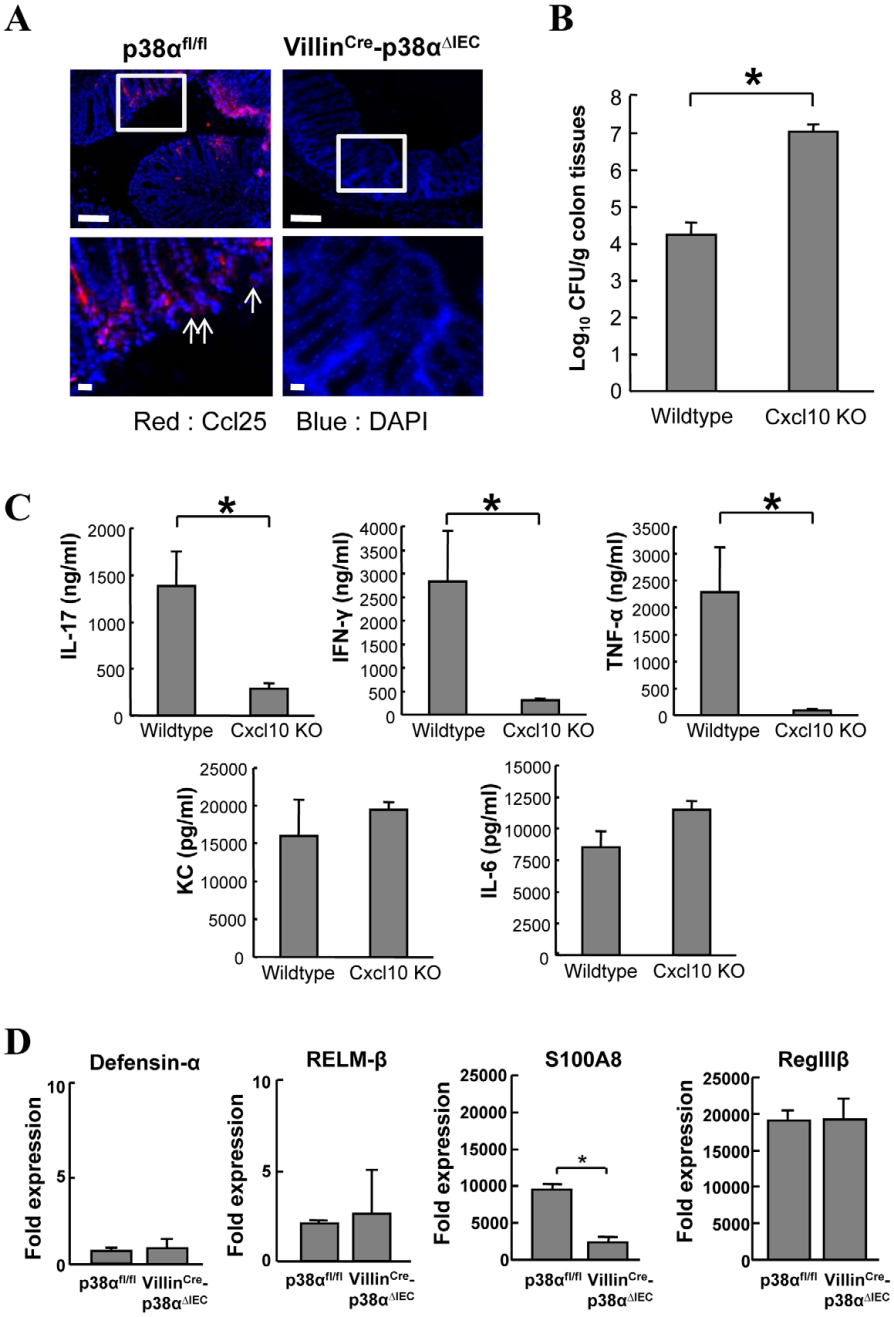
Analysis of chemokines Ccl25 and Cxcl10, and antimicrobial gene expression. **A**, Ccl25 expression in the colon mucosa of uninfected p38α^fl/fl^ and p38α^fl/fl^ or Villin^Cre^-p38α^ΔIEC^ mice at 1 week after infection, determined by immunofluorescent staining (red). Nuclei were counterstained with DAPI (blue). Data are representative of three independent experiments. **B**, Impaired bacteria clearance in *C. rodentium*-infected Cxcl10 knockout mice. Colon tissues from 2-week *C. rodentium*-infected wildtype or Cxcl10 knockout mice were obtained and bacterial CFU was determined. Asterisk, p<0.05. Error bars indicate s.d. (*n* = 3). **C**, *ex vivo* colon culture ELISA of IL-17, IFN-γ, TNF-α, KC, and IL-6 of *C. rodentium*-infected wildtype or Cxcl10 knockout mice. Asterisk, p<0.01. Error bars indicate s.d. (*n* = 3). **D**, Quantitative real-time PCR of antimicrobial gene expression in isolated IECs of p38α^fl/fl^ or Villin^Cre^-p38α^ΔIEC^ mice at 1 week after infection. Asterisk, p<0.05. Error bars indicate s.d. Data is representative of three experiments.

We further studied two chemokines, Ccl25 (also known as TECK) and Cxcl10 (also known as IP-10), to evaluate whether their associated impairment in expression following p38α deletion might contribute to the phenotype of the Villin^Cre^-p38^ΔIEC^ mice. Analyzing *C. rodentium* infection in Caco-2 cells revealed that the expression of Ccl25 and Cxcl10 was directly induced by *C. rodentium* in a p38-dependent manner (Supplementary Fig. S9). Immunofluorescence revealed lower induction of Ccl25 in the colonic epithelial cells of Villin^Cre^-p38^ΔIEC^ mice after *C. rodentium* infection in comparison with p38α^fl/fl^ mice (Fig. 6A). Since Ccl25 is one of the CD4^+^ T cell-recruiting molecules [21], the loss of Ccl25 induction should contribute to the impaired recruitment of CD4^+^ T cells into the colonic mucosa of Villin^Cre^-p38^ΔIEC^ mice. Cxcl10 is a chemoattractant for monocytes/macrophages, T cells, NK cells, and dendritic cells, and it promotes T cell adhesion to endothelial cells. Since mice lacking this gene are available, we assessed its role in host defense. Analysis of *C. rodentium*-infection in Cxcl10 knockout mice revealed that bacterial clearance was impaired in *C. rodentium*-infected Cxcl10 knockout mice (Fig. 6B). As observed in Villin^Cre^-p38^ΔIEC^ mice, the induction of IL-17 and IFN-γ was impaired in Cxcl10 knockout mice (Fig. 6C). Unlike Villin^Cre^-p38^ΔIEC^ mice, the induction of KC and IL-6 was not affected, but TNF induction was inhibited by Cxcl10 deletion (Fig. 6C). Despite the similarities in phenotype between Villin^Cre^-p38^ΔIEC^ and Cxcl10 mice in response to *C. rodentium*, the differences were also anticipated, since Cxcl10 induction should be only part of the mechanism of p38α-mediated host-defense against *C. rodentium* infection.

Parasite-induced RELM-β and Gob5 in intestinal epithelial cells can be blocked by deletion of IKK-β[10], [11], whereas their expression was not affected by deletion of p38α (Fig. 6D and data not shown). In addition, expression of S100A8, an antibacterial protein, was lower in Villin^Cre^-p38^ΔIEC^ mice, while other antibacterial peptides, Defensin-α and RegIIIβ, were similarly expressed (Fig. 6D). These data again show that intestinal epithelial p38α and NF-κB have different functions in initiating immune responses from the mucosal surface of the intestinal tract, and p38α is required for chemokine expression in the intestinal epithelial cells, which have crucial roles in recruiting immune cells into the colon mucosa upon bacterial infection.

## Discussion

Recent findings have shown that blocking NF-κB signaling in intestinal epithelial cells leads to dramatic impairments in mucosal immune responses and/or dysregulated intestinal epithelial cell integrity [10], [11]. Because MAP kinases represent another major inflammatory pathway in the gut that has not been explored in detail, we addressed the role of p38α in intestinal epithelial cells in the context of an A/E bacterial infection using mice with p38α-specific deletion in intestinal epithelial cells. Here we reported that, unlike NF-κB pathway deletion, p38α deletion is not involved in the dysregulation of immune cell function or epithelial cell integrity, but it is involved in the dysregulated chemokine expression and subsequent immune cell recruitment to the infected lesions.

While the host immune response against *C. rodentium* is still not fully characterized, it is known to involve T_H_1 and/or T_H_17 cells [7], [9]. The importance of CD4^+^ T cells in this infection has been demonstrated by the fact that *C. rodentium* infection is fatal in mice lacking CD4^+^ T cells [5]. While we do not fully understand how localized CD4^+^ T cells recruited to the infected lesion are involved in fighting this bacterial infection, previous studies have implicated T cells in much of the tissue pathology seen during infection, including mucosal hyperplasia [6], and inflammation that may limit *C. rodentium* survival/colonization at the mucosal surface. Similarly, CD4^+^ T cell dependent IgG antibodies are required for survival and clearance of this pathogen [5]. Therefore, the observed defect in CD4^+^ T cell recruitment to the intestinal mucosa in Villin^Cre^-p38^ΔIEC^ mice is likely to be the cause of their impaired defense against *C. rodentium.* This defect should also impair the “amplification cycles” of the immune response in the infected lesions, since the reduced immune cell recruitment is linked to the subsequent attenuation in cytokine production from epithelial cells. In fact, the reduced expression of S100A8, which is one of the antimicrobial proteins against *C. rodentium* and is regulated by IL-22 [20], was also detected in Villin^Cre^-p38^ΔIEC^ epithelial cells after infection.

It should be noted that many bacterial pathogens aside from *C. rodentium* induce p38α phosphorylation in intestinal epithelial cells, usually as an innate driven response to their products such as flagellin [16]. Thus it will be interesting to see whether the critical role played by p38 signaling in the host response to *C. rodentium* can be replicated in other infection models. Moreover the importance of p38α in the host response to A/E pathogens is further highlighted by the fact that these pathogens are known to suppress p38 activation in infected IEC, through the actions of their type III secretion systems [16]. While the mechanisms and bacterial effector proteins involved in this subversion have yet to be identified, our current studies suggest these actions may prove to be protective to the pathogen by limiting the recruitment of T cells to the gut.

Although we show here that p38α deletion in intestinal epithelial cells is linked with reduced chemokine expression and reduced immune cell recruitment to the lesions, we cannot fully exclude the possibility that for some pathogens, p38α is also related to the impairment of other immune functions in intestinal epithelial cells, as differential blocking of NF-κB pathway components during infection by different pathogens induced different host reactions. Nonetheless, our study defines a crucial role for p38α in intestinal epithelial cells for triggering the host immune responses in the gastrointestinal tract. The different function of different signaling pathways must be taken into consideration in the development and application of anti-inflammatory agents.

## Methods

### Mice

#### Ethics statement

Animal experiments were performed according to the guidelines of the Animal Care and Use Committee of The Scripps Research Institute (ARC-20NOV6) and Xiamen University (approval date 1/14/2009).

p38α^fl/fl^ mice were described previously [15]. Villin^Cre^ mice were obtained from The Jackson Laboratory (Bar Harbor, ME). All mice were backcrossed onto the C57Bl/6 strain for more than ten generations. 6–8 week old mice were used for the experiments and the littermate mice carrying the *loxP*-franked alleles but not expressing Cre recombinase were used as wild-type controls. Cxcl10-deficient mice were obtained from The Jackson Laboratory (Bar Harbor, ME).

### Bacterial infection and antigen preparation

2×10^9^ CFU *C. rodentium* strain DBS 100 (ATCC 51459; American Type Culture Collection, Manassas, VA) in a total volume of 200 µl was orally inoculated into each mouse after fasting for 8 hours. The concentration of bacteria was measured by absorbance at optical density 600, and was serially diluted and seeded on a MacConkey agar (Difco Laboratories, Sparks, MD) plate to confirm the CFU administered. Body weight changes were monitored daily. *Citrobacter* antigen was prepared as previously described [22]. Briefly, *C. rodentium* culture was washed with ice-cold PBS and sonicated on ice. The homogenate was then centrifuged at 4°C for 30 min. Supernatants were collected and sterilized by 0.22 µm filtration, and protein concentrations were determined.

### Tissue collection, bacterial DNA quantitation, colony-forming unit counts, and immunohistochemistry

After euthanizing mice, entire colon and mesenteric lymph nodes were removed under aseptic conditions. The terminal 0.5-cm piece of the colon was weighed, homogenized, serially diluted, and plated in triplicate on MacConkey agar plates to quantify bacterial numbers. To measure the bacterial 16s rDNA, tissue DNA was prepared from the colon of the infected mice using DNeasy Blood & Tissue Kit (Qiagen, Valencia, CA). Quantitative real-time PCR was performed using 50 ng of DNA and bacterial universal primers for r16 (forward; TCCTACGGGAGGCAGCAGT, and reverse; GGACTACCAGGGTATCTAATCCTGTT). GAPDH level was measured as a reference. The adjacent 0.5-cm piece was fixed in 10% formalin for H&E and *C. rodentium* staining, or frozen in optimal cutting temperature media (Tissue-Tek, Elkhart, In) for staining of other cell markers and Ccl25 expression. Immunostaining was performed as described previously [23]. Rabbit anti-*Citrobacter* antibodies (kindly provided by Dr. David B. Schauer; MIT) were used to identify adherent *C. rodentium* [3], and Rabbit anti-mouse Ccl25 antibodies were purchased from Santa Cruz Biotechnology (Santa Cruz, CA) to identify Ccl25 expression. Alexa Fluor 594 conjugated anti-rabbit IgG (Molecular Probes) was used for visualization. Alexa 488 conjugated CD4 (GK1.5), Gr-1 (RB6-8C5), and CD11c (N418) antibodies were purchased from BioLegend (San Diego, CA). Unconjugated anti-CD4 (GK1.5) and anti-IL-17 antibodies were obtained from Santa Cruz Biotechnology (Santa Cruz, CA). Slides were mounted using VectaShield with DAPI (Vector Labs, Burlingame, CA). The *in situ* Cell Death Detection Kit (Roche, Mannheim, Germany) was used for TUNEL staining (TdT-mediated dUTP nick end-labeling). The degree of inflammatory cell infiltration was assessed by a histological score. The score was defined as a scale of 0–3 as follows: inflammatory cell infiltration; 0 =  occasional inflammatory cells in the lamina propria; 1 =  increased number of inflammatory cells in the lamina propria; 2 =  confluent inflammatory cells, extending into the submucosa; 3 =  transmural extension of the infiltrate.

### Isolation of primary colon epithelial cells

Colon epithelial cells were isolated using a modified rapid low-temperature method as described previously [24]. Briefly, the entire colon was removed and washed with ice-cold PBS. After dividing the intestine into 2–3 mm long fragments and transferring them into chelating buffer (27 mM trisodium citrate, 5 mM Na_2_PO_4_, 96 mM NaCl, 8 mM KH_2_PO_4_, 1.5 mM KCl, 0.5 mM DTT, 55 mM D-sorbitol, 44 mM Sucrose, 6 mM EDTA, 5 mM EGTA, pH 7.3) for 45 min. at 4°C, epithelial cells were then dissociated by repeated vigorous shaking. Tissue debris was removed by a cell-strainer (100 µm) and colon epithelial cells were collected by centrifugation at 150×g for 10 min. at 4°C. The viability of colon epithelial cells was confirmed by trypan blue staining and processed for protein or RNA extraction.

### Immunoblot analysis

Total cell extracts from colonic epithelial cells were analyzed by SDS-polyacrylamide gel electrophoresis and transferred to polyvinylidene difluoride membranes (Hybond-P; Amersham Pharmacia Biotech, Buckinghamshire, UK), followed by immunoblotting with anti-p38α, anti-phosphorylated p38 (Cell Signaling Technology, Danvers, MA), and anti-GAPDH (Chemicon, Temecula, CA) antibodies. The bound antigens were detected using SuperSignal West Femto Maximum Sensitivity Substrate (Pierce, Rockford, IL).

### Dendritic cell isolation, restimulation, and flow cytometry analysis

Mesenteric lymph nodes were aseptically prepared and dendritic cells were isolated by positive selection using CD11c^+^ MACS microbeads (Miltenyi Biotec). Cells were stained with anti-CD11c-APC, CD11b-PerCP, and CD8α-FITC antibodies (eBioscience). Intracellular TNF staining was performed using Fixation/Permeabilization buffers and anti-TNF-PE antibodies (eBioscience). Stained samples were analyzed using a FACScalibur flow cytometer and FlowJo software.

Lymphpcytes isolated from mesenteric lymph nodes were cultured at a concentration of 5×10^6^ cells/ml and restimulated with 50 µg/ml of *Citrobacter* antigen for 48 hours. Culture supernatants were prepared to measure the IL-17 and IFN-γ concentrations by ELISA (R&D systems).

### Lamina propria lymphocyte preparation and flow cytometry analysis

The colon was removed and opened longitudinally, then washed with ice-cold PBS to remove debris. The tissue was then cut into small pieces (∼1 cm) and further incubated for 30 min. at 37°C with gentle shaking in HBSS with 1 mM DTT and 2% FCS, and the supernatant was removed. The colon tissue was further incubated in HBSS with 1 mM EDTA and 2% FCS for 30 min. at 37°C with gentle shaking. Tissue was collected and further cut into smaller pieces, and digested with 0.5 mg/ml collagenase type IV (Sigma-Aldrich. St. Louis, MO) at 37°C with gentle shaking for 2 hrs. Cells were washed in HBSS twice and passed through a 40 µm cell strainer. Whole colon cells were resuspended in RPMI-1640 medium supplemented with 10% FBS and antibiotics, and treated with PMA and ionomycin for 6 hours. Intracellular staining of cytokines was performed using Cytofix/Cytoperm Fixation/Permeabilization Solution kit (BD Bioscience, San Jose, CA). Cells were harvested and stained with anti-CD4-PE and anti-IL-17-APC antibodies to measure the infiltration of CD4 cells and the expression of IL-17 in the whole colon. Lamina propria cells were harvested by discontinuous 40%/80% Percoll gradient centrifugation of whole colon cells. After centrifugation, cells in the interface were collected and washed twice in HBSS. After 6 hours of PMA and ionomycin treatment, cells were harvested and stained with anti-CD3-FITC, anti-CD4-PE, anti-IFN-γ-PerCP, and anti-IL-17-APC antibodies for flow cytometry analysis.

### Colon culture and cytokine measurement

Entire colons were removed and cultured at 37°C for 24 hours as described previously [20]. Supernatants were collected and IL-17, IFN-γ, IL-22, KC, IL-6, and TNF levels were analyzed by ELISA (R&D systems).

### Cell culture and *in vitro* Citrobacter infection

Caco-2 human colonic epithelial cell lines (ATCC) were grown in DMEM supplemented with 10% FBS without antibiotics. Seven days after reaching confluency, the cells were infected with *C. rodentium* at a multiplicity of infection of 50. After 4 hours of incubation, as described previously [16], the cells were washed and RNA was extracted to assay the cell responses. In the case of using a p38 inhibitor, SB203580 (Calbiochem, San Diego, CA) was added at 5 nM for 1 hour prior to infection.

### RNA isolation, microarray analyses, and real-time reverse transcribed PCR

Total RNA from isolated colonic epithelial cells and Caco-2 cells was isolated using Trizol reagent (Invitrogen, Carlsbad, CA) and analyzed by chemokine & receptor oligomicroarrays (Oligo GEArray OMM-022 or OHS-022; SABiosciences, Frederick, MD) according to the manufacturers' instructions. Colon tissues from Citrobacter-infected or uninfected mice were obtained and total RNA was prepared and cDNA was synthesized by reverse transcription. Microarray data analyses were performed using GEArray Expression Analysis Suite version 2.0 software, according to the manufacturer's instructions (SABiosciences). The expression threshold was determined to be when the average density of the spot is more than the mean value of the local backgrounds of the lower 75^th^ percentile of all spots. Quantitative real-time PCR was performed using a TaqMan gene expression system with Sybr Green (Applied Biosystems, Foster City, CA). The primer sequences are listed in Table S2. All values were normalized to the level of the house keeping gene GAPDH messenger RNA, and relative expression was calculated according to the *ΔΔC_T_* method.

### Statistical analysis

The statistical significance of the differences between the two groups was determined using the Student's *t* test when variances were equal, or using the Welch's *t* test when variances were unequal.

## Supporting Information

Figure S1 Sustained body weight loss in Villin^Cre^-p38α^ΔIEC^ mice after *C. rodentium* infection. 2×10^9^ CFU/mouse *C. rodentium* was inoculated into p38α^fl/fl^ (*n* = 18) and Villin^Cre^-p38α^ΔIEC^ mice (*n* = 19) orally. Body weight changes were monitored daily.
(0.11 MB TIF)

Figure S2 *C. rodentium* CFU recovered from distal colon tissues and feces of individual p38α^fl/fl^ and Villin^Cre^-p38α^ΔIEC^ mice at 1, 2, and 3 weeks (a) or 7, 10, and 14 days (b) after inoculation. The data shown are in logarithmic scale and from one experiment, representative of five. The transverse bar is the detection limit. Asterisk, p<0.05. Error bars indicate s.d. (*n* = 6).
(0.15 MB TIF)

Figure S3 No bacterial invasion into the colon mucosa in p38α^fl/fl^ and Villin^Cre^-p38α^ΔIEC^ mice. Immunofluorescence staining of *C. rodentium* by anti-*C. rodentium* antibodies (red) in the colon segments two weeks after infection. Close images of the mucosa denoted by the boxes in the upper panels (same of the Fig.1D) are shown in the lower panels. Scale bar, 100 µm.
(0.75 MB TIF)

Figure S4 Expression of TNF-α by the CD11c+CD11b+CD8α- (CD11b+, **A**), CD11c+CD11b-CD8α+ (CD8α+, **B**), or CD11c+CD11b-CD8α- (double negative, C) DC subset population of the draining mesenteric lymph nodes of p38α^fl/fl^ or Villin^Cre^-p38α^ΔIEC^ mice at 1 and 2 weeks after infection. Data are representative of three independent experiments (*n* = 3). Expression profiles of TNF in the cells from uninfected p38α^fl/fl^ or Villin^Cre^-p38α^ΔIEC^ mice were similar. Expression of TNF uninfected p38α^fl/fl^ mouse is shown as control.
(0.24 MB TIF)

Figure S5 Expression of IL-17 and IFN-γ in mesenteric lymph node lymphocytes. After 2 weeks of *C. rodentium* infection, lymphocytes from mesenteric lymph nodes were obtained from p38α^fl/fl^ and Villin^Cre^-p38α^ΔIEC^ mice, and stimulated with PMA (10 ng/ml) and ionomycin (1 µm) for 6 hours. Cells were harvested and stained with anti-CD3 and anti-CD4 antibodies, and then further stained with anti-IL-17 and anti-IFN-γ antibodies to detect the expression of intracellular IL-17 and IFN-γ by FACS analysis.
(0.34 MB TIF)

Figure S6 The same as Supplementary Figure S5 except lamina propria lymphocytes were used.
(0.34 MB TIF)

Figure S7 Only a few scattered immune cells infiltrated into the mucosa were detected at 1 week after infection in p38α^fl/fl^ and Villin^Cre^-p38α^ΔIEC^ mice. Cell infiltration in the distal colon mucosa of p38α^fl/fl^ or Villin^Cre^-p38α^ΔIEC^ mice at 1 week after infection, determined by immunofluorescent staining for CD4, CD11c, and Gr-1 (green). Nuclei were counterstained with DAPI (blue). Scale bar, 100 µm (low magnification), 30 µm (high magnification). Data are representative of 2–4 independent experiments (*n* = 4).
(2.47 MB TIF)

Figure S8 CD4+ T cell infiltration in the distal colon mucosa is greater in p38α^fl/fl^ than those in Villin^Cre^-p38α^ΔIEC^ mice. CD4+ T cell infiltration in the colon mucosa at 2 weeks after infection was determined by immunofluorescent staining (green). Scale bar, 30 µm. Nuclei were counterstained with DAPI (blue).
(1.07 MB TIF)

Figure S9 **A, B,** Linear scatter plot of gene expression in Caco-2 cells after *in vitro* infection with and without a p38 inhibitor. Caco-2 cells were infected with *C. rodentium in vitro* at 50 multiplicity of infection for four hours (**A**). p38 inhibitor, SB203580, was added at 5 nM for 1 hour prior to infection (**B**). Gene expression was determined by chemokine & receptor oligomicroarrays in comparison with that of uninfected control Caco-2 cells. Each gene in the microarray is represented by a point in logarithmic scale. The genes with more than 2 fold increase or less than 0.5 fold decrease are represented as red or green, respectively.
(0.22 MB TIF)

Table S1 Quantitation of *Citrobacter rodentium* infection by qPCR.
(0.11 MB PPT)

Table S2 The sequences of quantitative PCR primers.
(0.12 MB PPT)
