# Supplementary figures and images for: Epithelial p38α Controls Immune Cell Recruitment in the Colonic Mucosa
Source: PLoS Pathog. 2010 Jun 3;6(6):e1000934. doi: 10.1371/journal.ppat.1000934 (PMC2880565; doi:10.1371/journal.ppat.1000934)

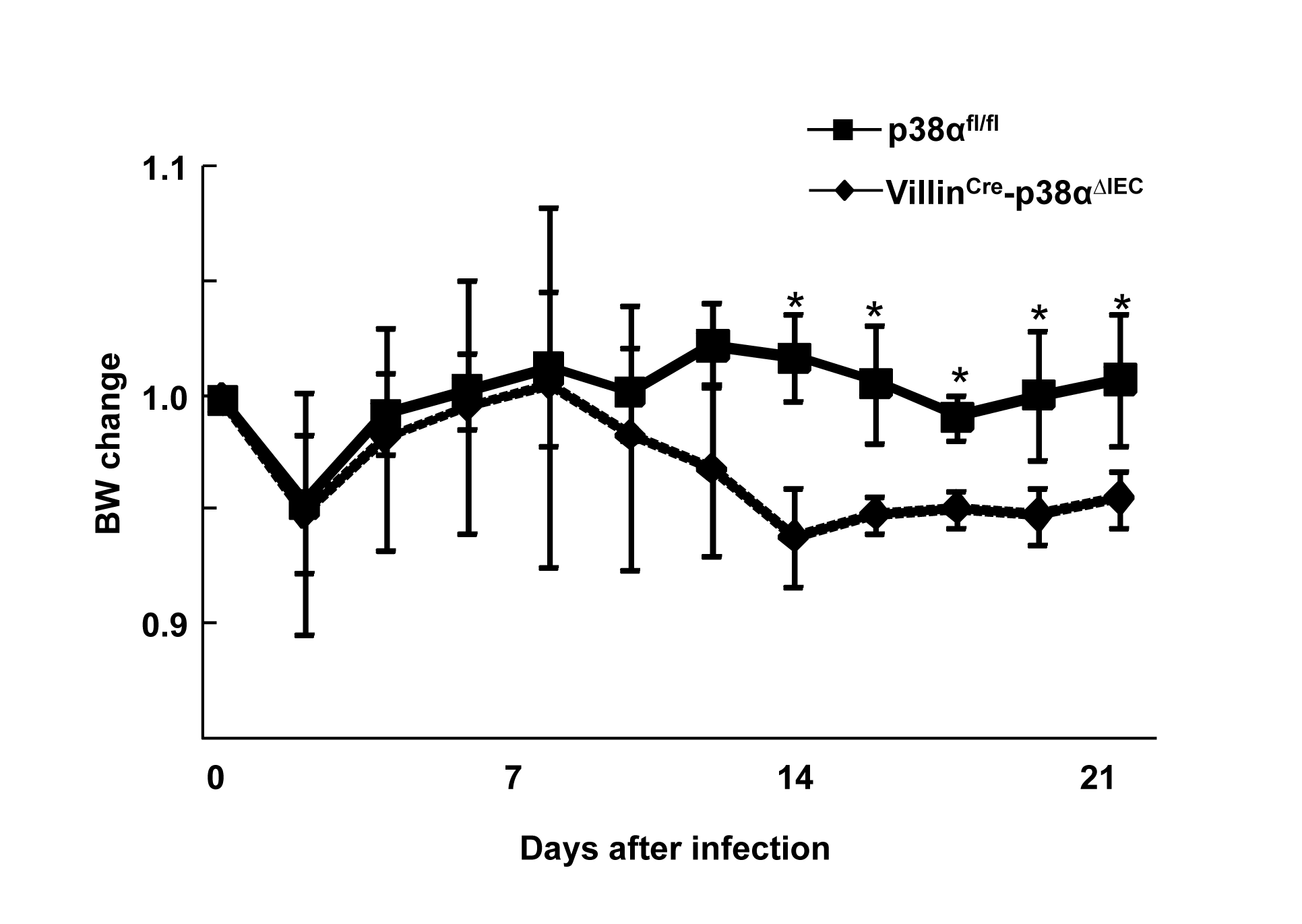

Supplement: Figure S1 — Sustained body weight loss in VillinCre-p38αΔIEC mice after C. rodentium infection. 2×10^9^ CFU/mouse C. rodentium was inoculated into p38αfl/fl (n = 18) and VillinCre-p38αΔIEC mice (n = 19) orally. Body weight changes were monitored daily. (0.11 MB TIF) [file ppat.1000934.s001.tif]

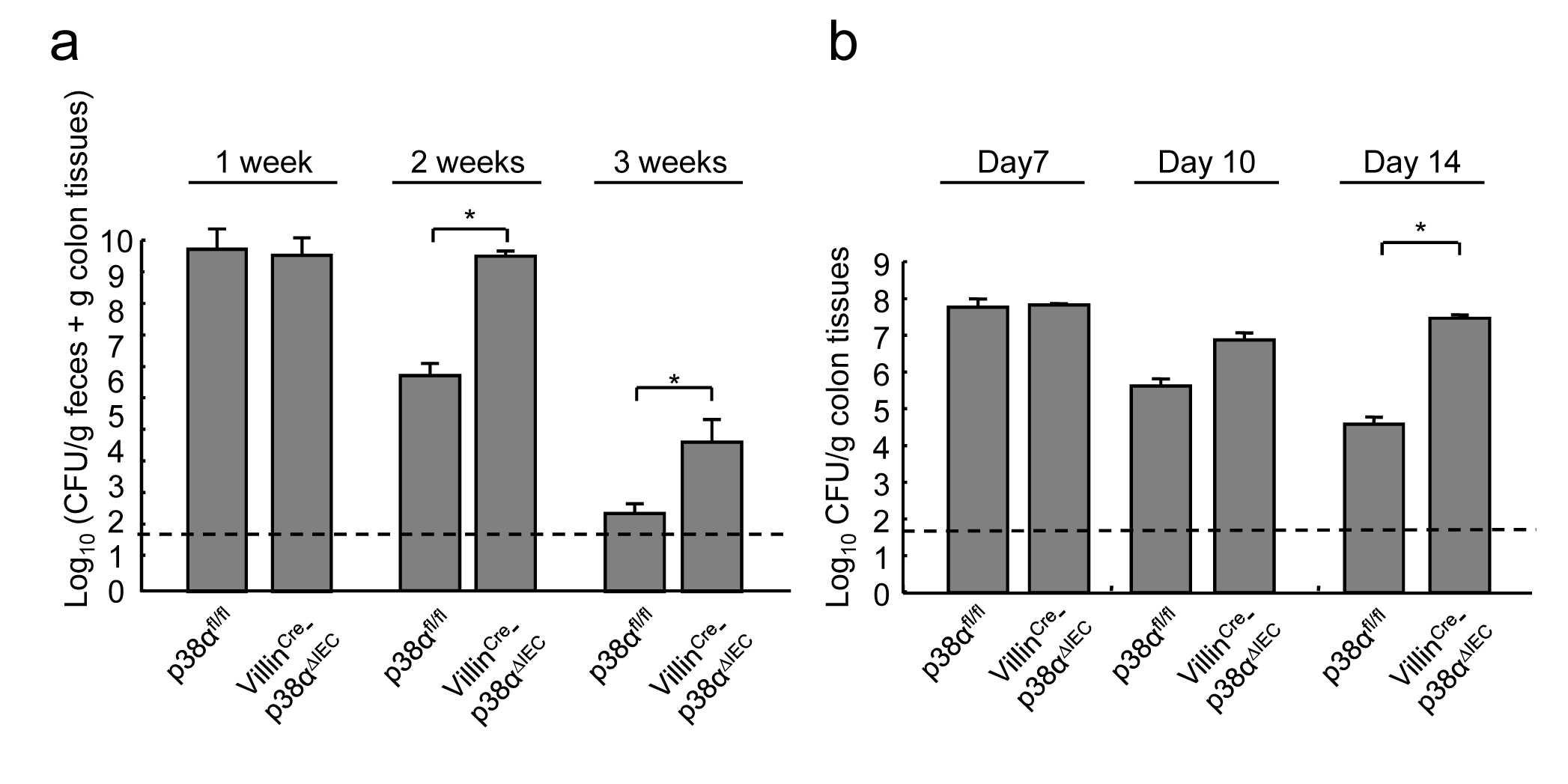

Supplement: Figure S2 — C. rodentium CFU recovered from distal colon tissues and feces of individual p38αfl/fl and VillinCre-p38αΔIEC mice at 1, 2, and 3 weeks (a) or 7, 10, and 14 days (b) after inoculation. The data shown are in logarithmic scale and from one experiment, representative of five. The transverse bar is the detection limit. Asterisk, p<0.05. Error bars indicate s.d. (n = 6). (0.15 MB TIF) [file ppat.1000934.s002.tif]

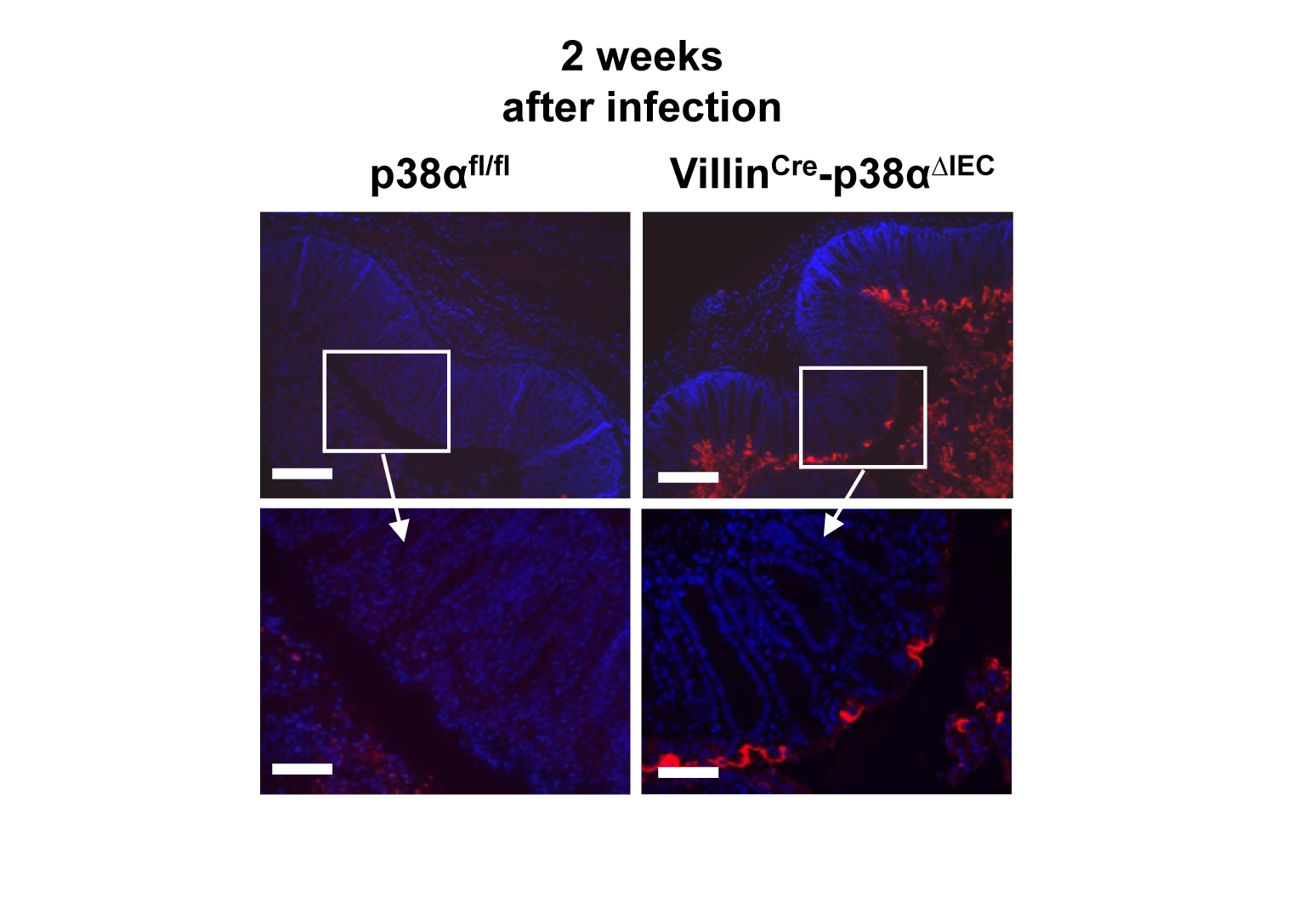

Supplement: Figure S3 — No bacterial invasion into the colon mucosa in p38αfl/fl and VillinCre-p38αΔIEC mice. Immunofluorescence staining of C. rodentium by anti-C. rodentium antibodies (red) in the colon segments two weeks after infection. Close images of the mucosa denoted by the boxes in the upper panels (same of the Fig.1D) are shown in the lower panels. Scale bar, 100 µm. (0.75 MB TIF) [file ppat.1000934.s003.tif]

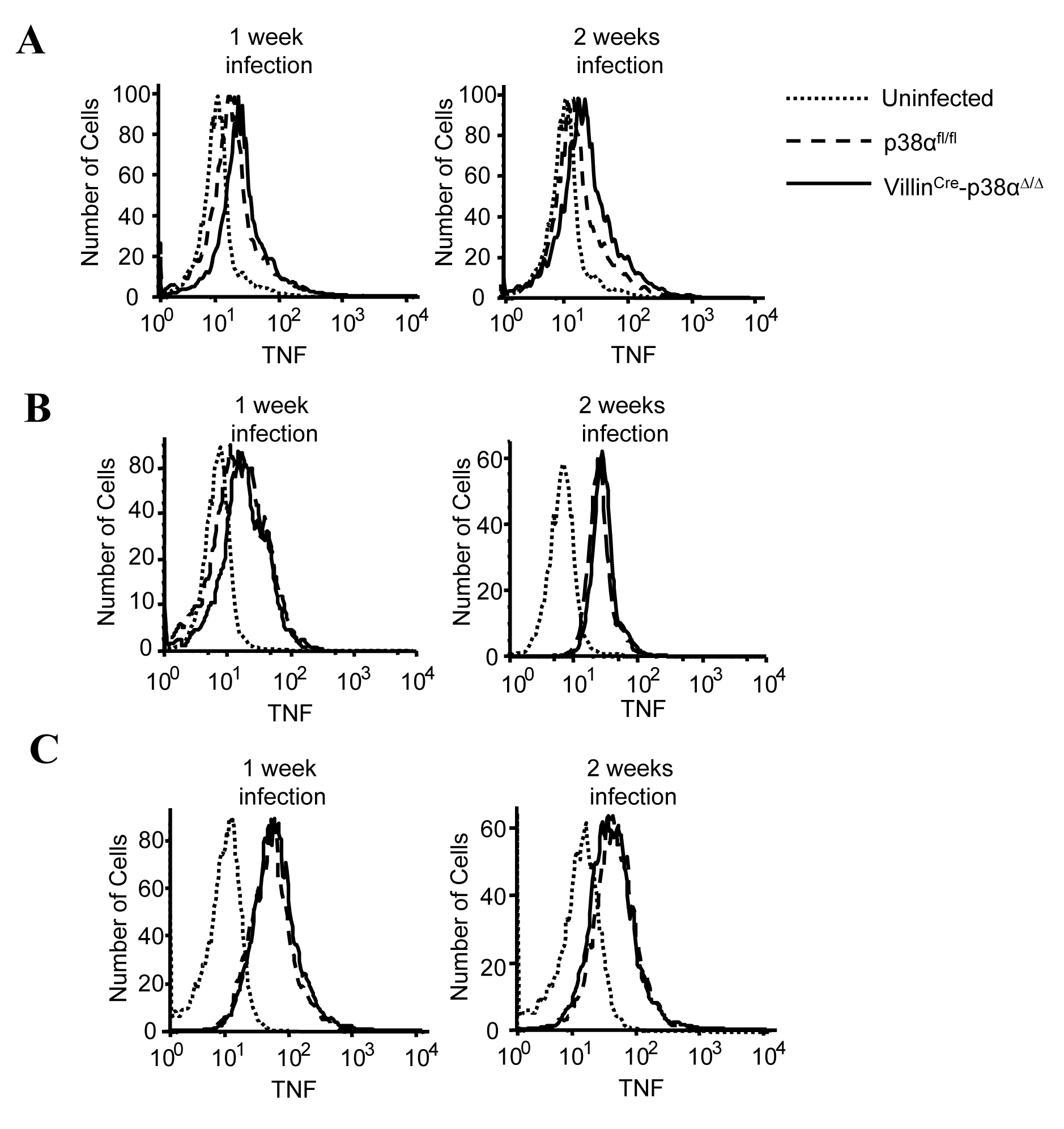

Supplement: Figure S4 — Expression of TNF-α by the CD11c+CD11b+CD8α- (CD11b+, A), CD11c+CD11b-CD8α+ (CD8α+, B), or CD11c+CD11b-CD8α- (double negative, C) DC subset population of the draining mesenteric lymph nodes of p38αfl/fl or VillinCre-p38αΔIEC mice at 1 and 2 weeks after infection. Data are representative of three independent experiments (n = 3). Expression profiles of TNF in the cells from uninfected p38αfl/fl or VillinCre-p38αΔIEC mice were similar. Expression of TNF uninfected p38αfl/fl mouse is shown as control. (0.24 MB TIF) [file ppat.1000934.s004.tif]

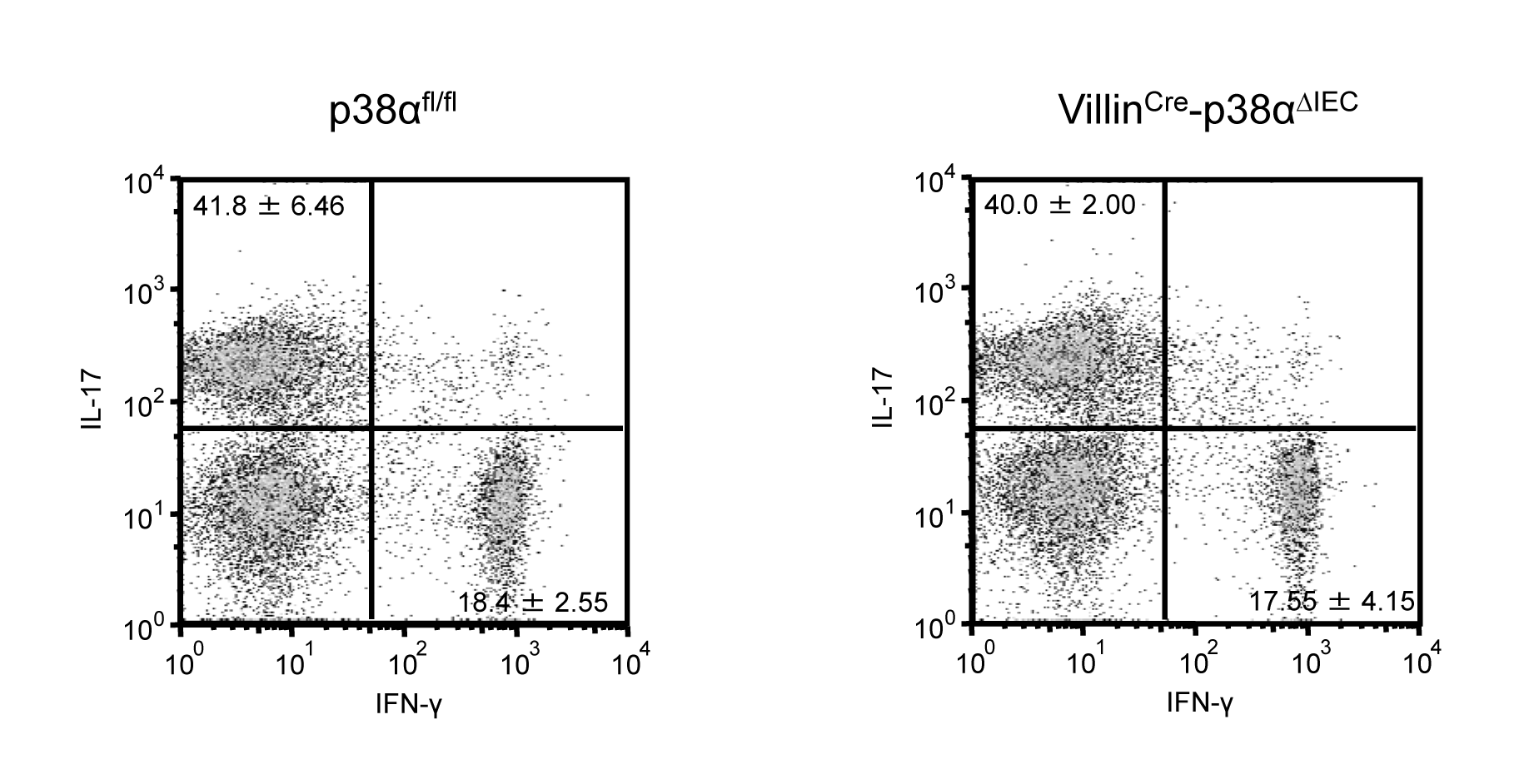

Supplement: Figure S5 — Expression of IL-17 and IFN-γ in mesenteric lymph node lymphocytes. After 2 weeks of C. rodentium infection, lymphocytes from mesenteric lymph nodes were obtained from p38αfl/fl and VillinCre-p38αΔIEC mice, and stimulated with PMA (10 ng/ml) and ionomycin (1 µm) for 6 hours. Cells were harvested and stained with anti-CD3 and anti-CD4 antibodies, and then further stained with anti-IL-17 and anti-IFN-γ antibodies to detect the expression of intracellular IL-17 and IFN-γ by FACS analysis. (0.34 MB TIF) [file ppat.1000934.s005.tif]

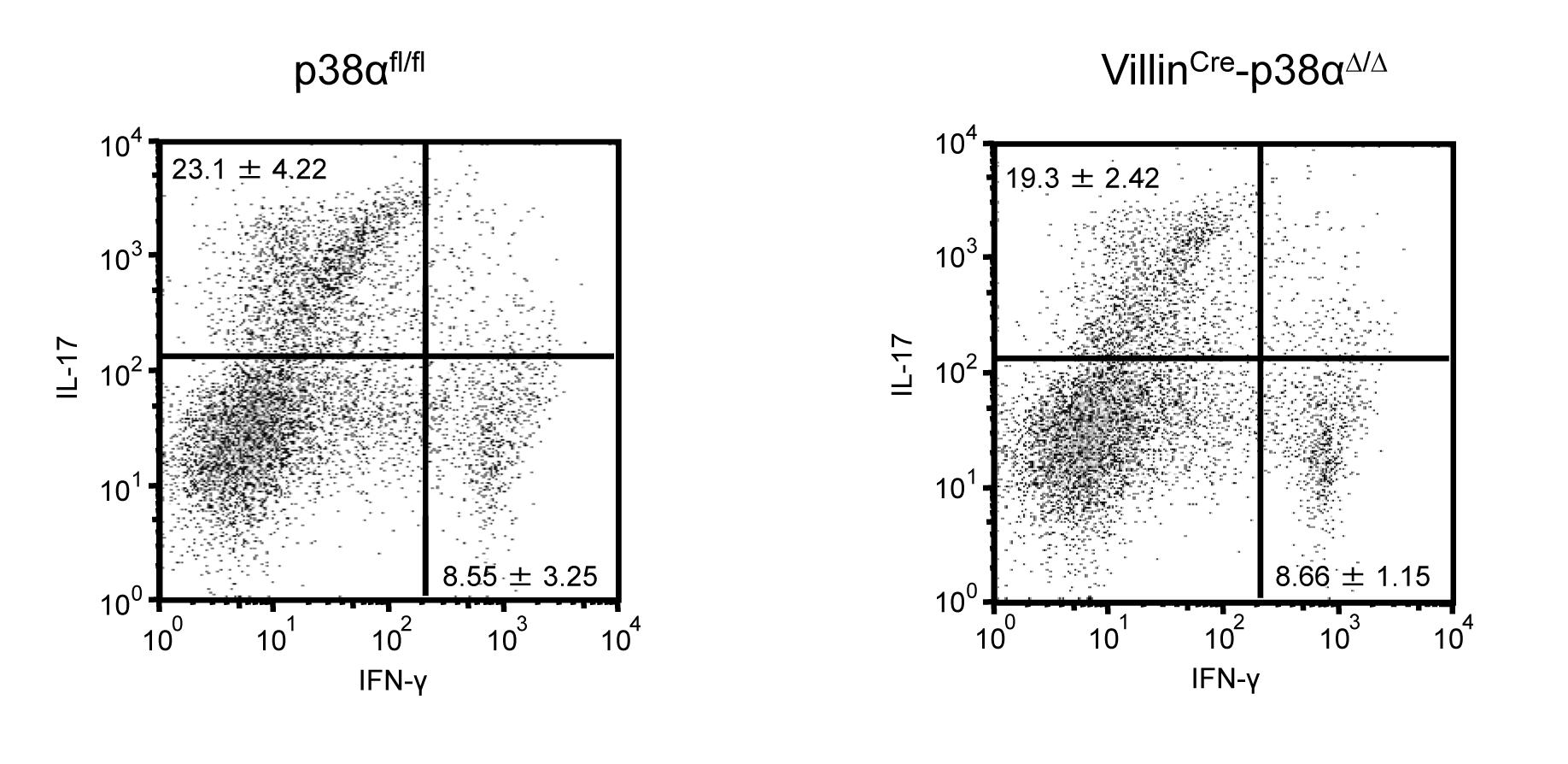

Supplement: Figure S6 — The same as Supplementary Figure S5 except lamina propria lymphocytes were used. (0.34 MB TIF) [file ppat.1000934.s006.tif]

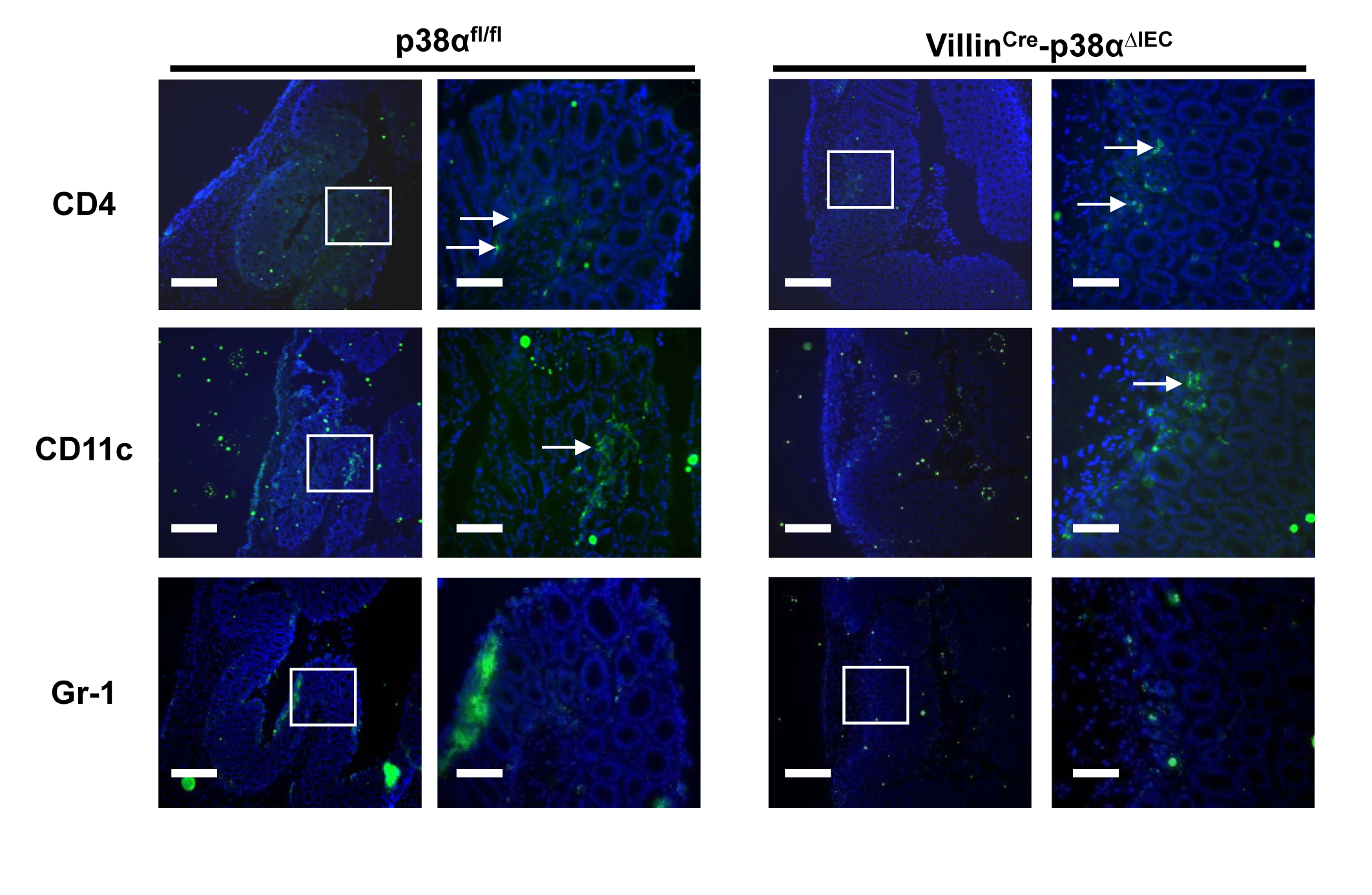

Supplement: Figure S7 — Only a few scattered immune cells infiltrated into the mucosa were detected at 1 week after infection in p38αfl/fl and VillinCre-p38αΔIEC mice. Cell infiltration in the distal colon mucosa of p38αfl/fl or VillinCre-p38αΔIEC mice at 1 week after infection, determined by immunofluorescent staining for CD4, CD11c, and Gr-1 (green). Nuclei were counterstained with DAPI (blue). Scale bar, 100 µm (low magnification), 30 µm (high magnification). Data are representative of 2–4 independent experiments (n = 4). (2.47 MB TIF) [file ppat.1000934.s007.tif]

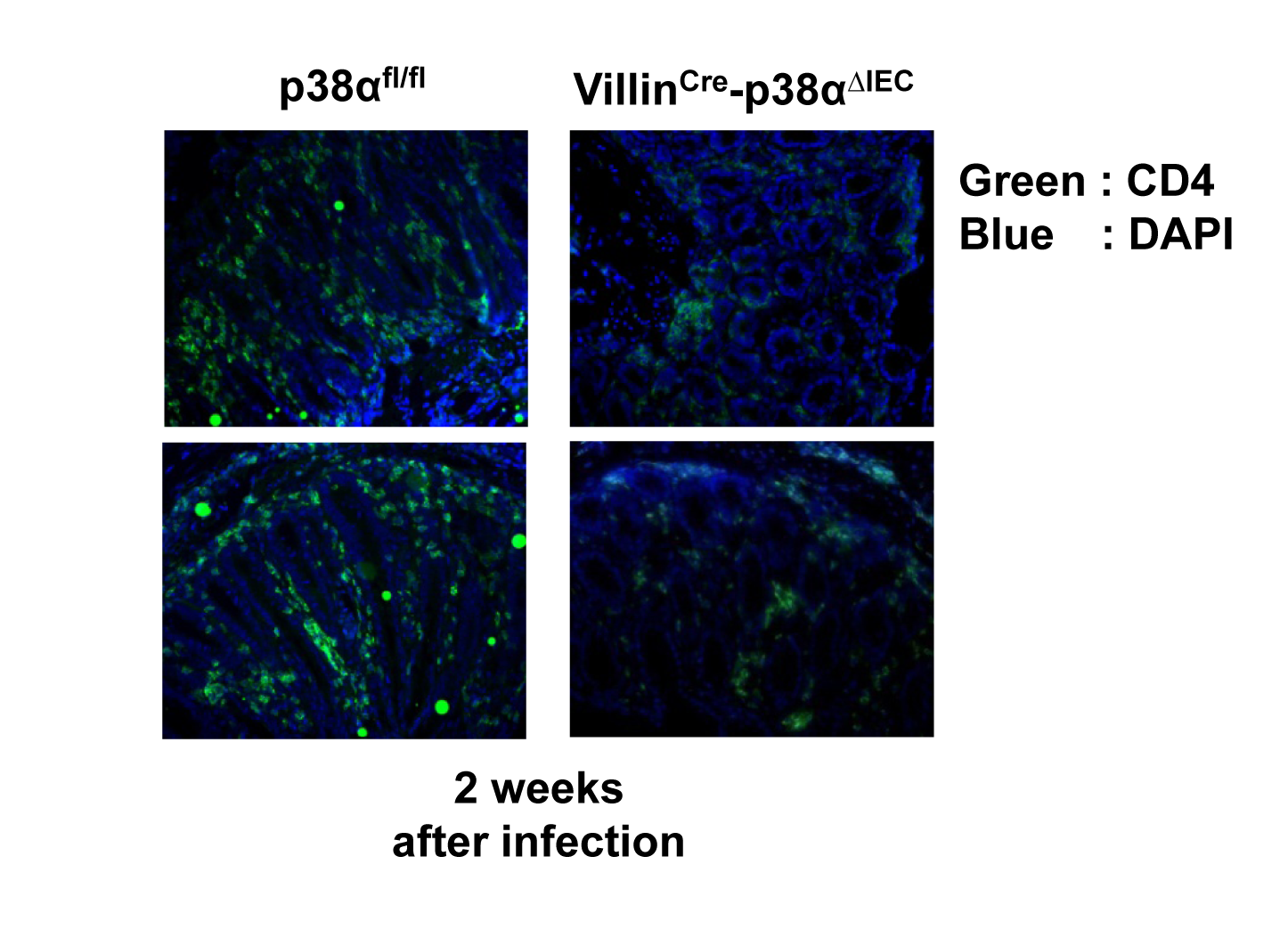

Supplement: Figure S8 — CD4+ T cell infiltration in the distal colon mucosa is greater in p38αfl/fl than those in VillinCre-p38αΔIEC mice. CD4+ T cell infiltration in the colon mucosa at 2 weeks after infection was determined by immunofluorescent staining (green). Scale bar, 30 µm. Nuclei were counterstained with DAPI (blue). (1.07 MB TIF) [file ppat.1000934.s008.tif]

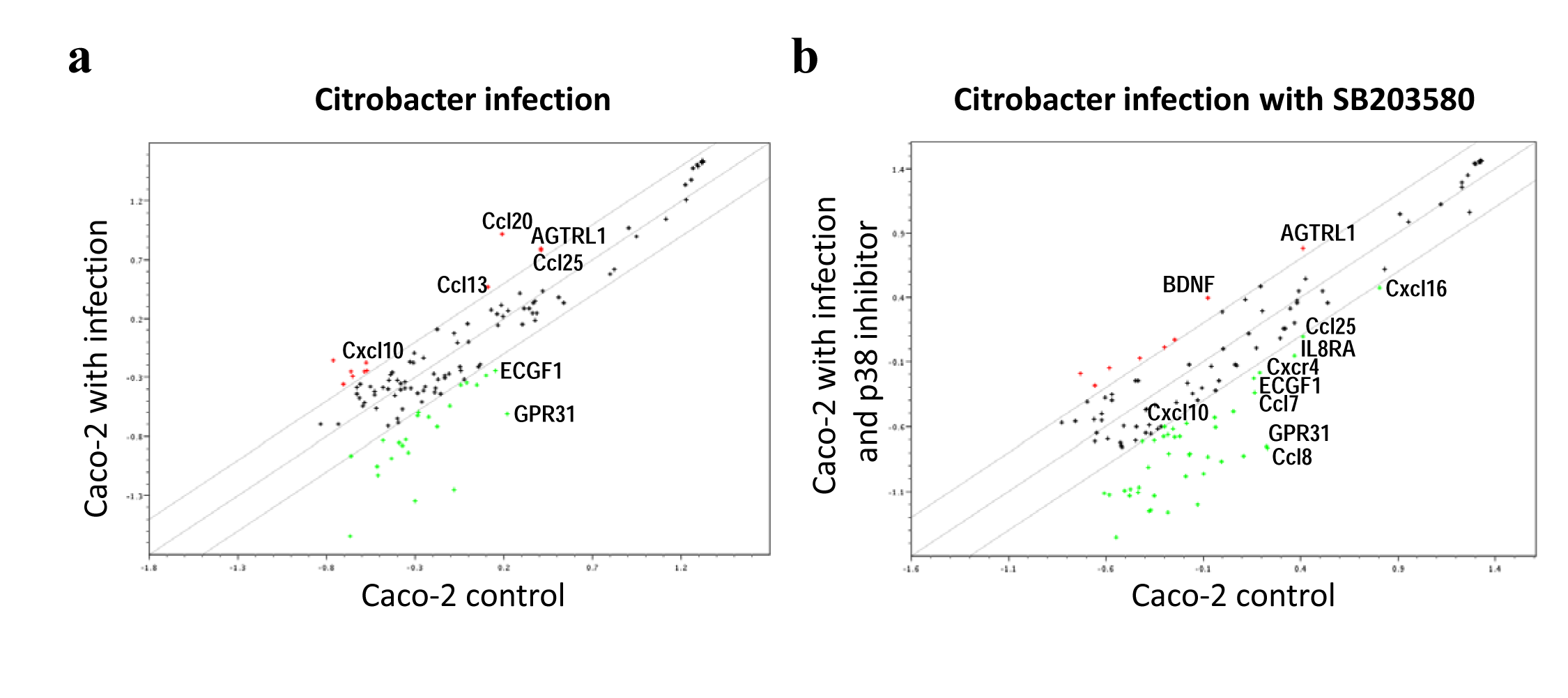

Supplement: Figure S9 — A, B, Linear scatter plot of gene expression in Caco-2 cells after in vitro infection with and without a p38 inhibitor. Caco-2 cells were infected with C. rodentium in vitro at 50 multiplicity of infection for four hours (A). p38 inhibitor, SB203580, was added at 5 nM for 1 hour prior to infection (B). Gene expression was determined by chemokine & receptor oligomicroarrays in comparison with that of uninfected control Caco-2 cells. Each gene in the microarray is represented by a point in logarithmic scale. The genes with more than 2 fold increase or less than 0.5 fold decrease are represented as red or green, respectively. (0.22 MB TIF) [file ppat.1000934.s009.tif]
