# Supplementary material for: Epithelial p38α Controls Immune Cell Recruitment in the Colonic Mucosa
Source: PLoS Pathog. 2010 Jun 3;6(6):e1000934. doi: 10.1371/journal.ppat.1000934 (PMC2880565; doi:10.1371/journal.ppat.1000934)
Supplement: Table S1 — Quantitation of Citrobacter rodentium infection by qPCR. (0.11 MB PPT) [file ppat.1000934.s010.ppt]

## Slide 1
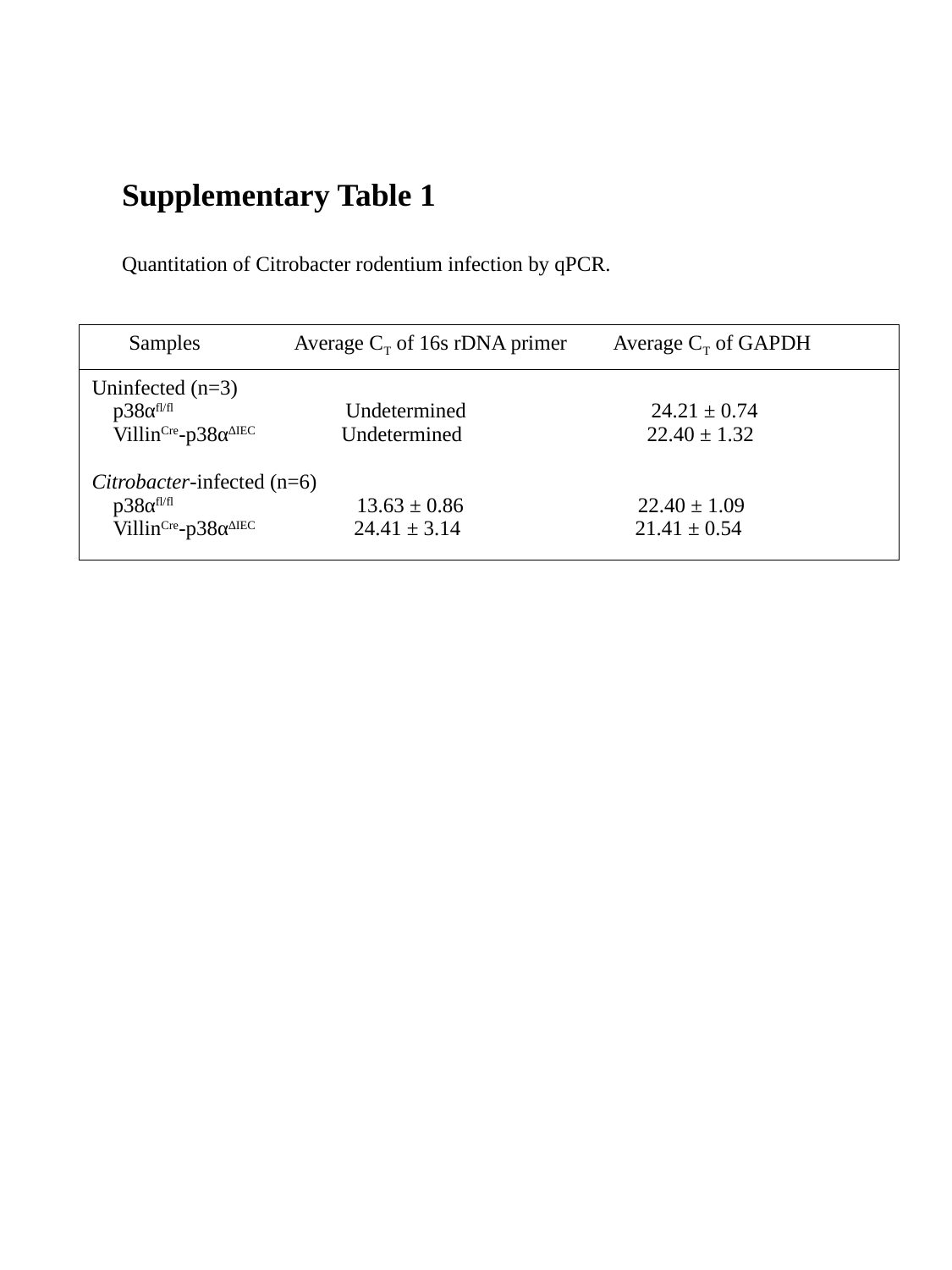

Supplementary Table 1
Quantitation of Citrobacter rodentium infection by qPCR.
| Samples Average CT of 16s rDNA primer Average CT of GAPDH |
| --- |
| Uninfected (n=3) p38αfl/fl Undetermined 24.21 ± 0.74 VillinCre-p38α∆IEC Undetermined 22.40 ± 1.32 Citrobacter-infected (n=6) p38αfl/fl 13.63 ± 0.86 22.40 ± 1.09 VillinCre-p38α∆IEC 24.41 ± 3.14 21.41 ± 0.54 |
