# Supplementary material for: Epithelial p38α Controls Immune Cell Recruitment in the Colonic Mucosa
Source: PLoS Pathog. 2010 Jun 3;6(6):e1000934. doi: 10.1371/journal.ppat.1000934 (PMC2880565; doi:10.1371/journal.ppat.1000934)
Supplement: Table S2 — The sequences of quantitative PCR primers. (0.12 MB PPT) [file ppat.1000934.s011.ppt]

## Slide 1
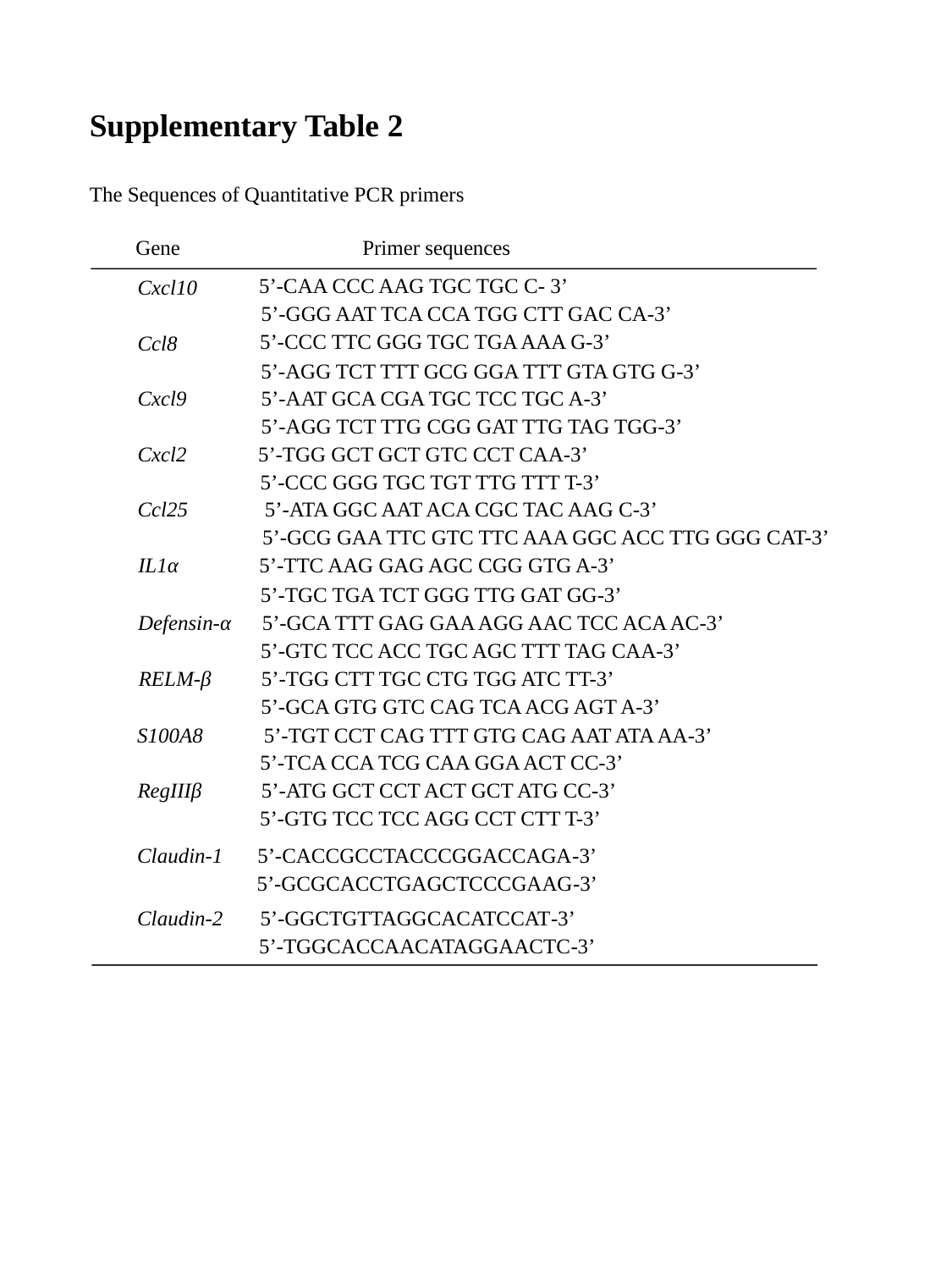

Supplementary Table 2
The Sequences of Quantitative PCR primers
Gene
Primer sequences
5’-CAA CCC AAG TGC TGC C- 3’
Cxcl10
5’-GGG AAT TCA CCA TGG CTT GAC CA-3’
5’-CCC TTC GGG TGC TGA AAA G-3’
Ccl8
5’-AGG TCT TTT GCG GGA TTT GTA GTG G-3’
Cxcl9
5’-AAT GCA CGA TGC TCC TGC A-3’
5’-AGG TCT TTG CGG GAT TTG TAG TGG-3’
Cxcl2
5’-TGG GCT GCT GTC CCT CAA-3’
5’-CCC GGG TGC TGT TTG TTT T-3’
Ccl25
5’-ATA GGC AAT ACA CGC TAC AAG C-3’
5’-GCG GAA TTC GTC TTC AAA GGC ACC TTG GGG CAT-3’
IL1α
5’-TTC AAG GAG AGC CGG GTG A-3’
5’-TGC TGA TCT GGG TTG GAT GG-3’
Defensin-α
5’-GCA TTT GAG GAA AGG AAC TCC ACA AC-3’
5’-GTC TCC ACC TGC AGC TTT TAG CAA-3’
RELM-β
5’-TGG CTT TGC CTG TGG ATC TT-3’
5’-GCA GTG GTC CAG TCA ACG AGT A-3’
S100A8
5’-TGT CCT CAG TTT GTG CAG AAT ATA AA-3’
5’-TCA CCA TCG CAA GGA ACT CC-3’
RegIIIβ
5’-ATG GCT CCT ACT GCT ATG CC-3’
5’-GTG TCC TCC AGG CCT CTT T-3’
Claudin-1
5’-CACCGCCTACCCGGACCAGA-3’
5’-GCGCACCTGAGCTCCCGAAG-3’
Claudin-2
5’-GGCTGTTAGGCACATCCAT-3’
5’-TGGCACCAACATAGGAACTC-3’
